# *Ab Initio* Investigation of Tetrel Bonds in Isolated Complexes Formed Between a Lewis Acid H_3_MX, M–O or M–S (M = Si, Ge, or Sn) and the Lewis Bases B = N_2_, CO, HCCH, PH_3_, C_2_H_4_, HCN, CS, HNC, NP, H_2_O, and NH_3_

**DOI:** 10.1021/acs.jpca.4c03438

**Published:** 2024-07-15

**Authors:** Ibon Alkorta, Anthony C. Legon

**Affiliations:** † Instituto de Química Médica (IQM-CSIC), Juan de la Cierva, 3, E-28006 Madrid, Spain; ‡ School of Chemistry, University of Bristol, Cantock’s Close, Bristol BS8 1TS, U.K.

## Abstract

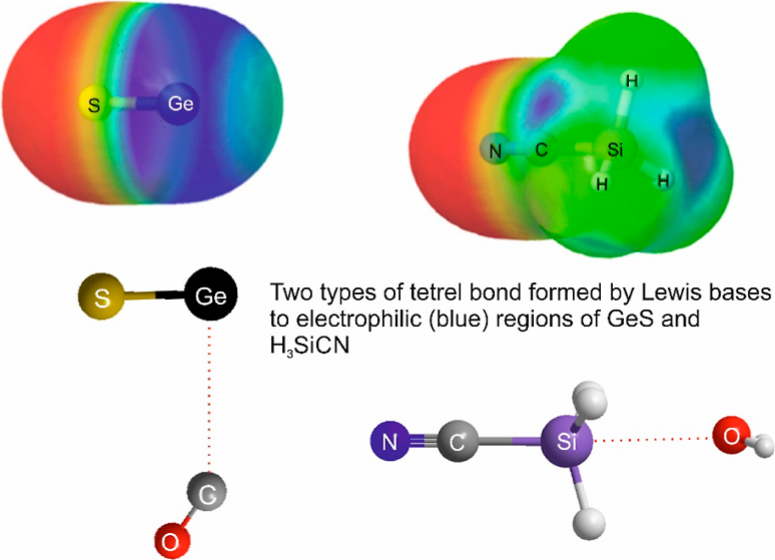

Isolated complexes of the type B⋯A in which the noncovalent interaction is a tetrel bond have been characterized by *ab initio* calculations at the CCSD(T)(F12c)/cc-pVDZ-F12 level. The Lewis bases B involved were N_2_, CO, HCCH, PH_3_, C_2_H_4_, HCN, CS, HNC, NP, H_2_O and NH_3_. Two types of Lewis acid A were examined, each containing one of the tetrel atoms M = Si, Ge or Sn, The Lewis acids in the first series were the H_3_MX (X = F, Cl, CN, H), in each of which the most electrophilic region was found to lie on the *C*_3_ axis of the C_3v_ molecules, near to the tetrel atom M. In the second series the Lewis acids were M–O and M–S. Graphs, consisting of calculated equilibrium dissociation energies *D*_e_ of each B⋯H_3_MX complex plotted against the nucleophilicities *N*_B_ of the Lewis bases B, were used to obtain the electrophilicity *E*_H3MX_ of each molecule H_3_MX (M = Si, Ge, Sn). The molecular electrostatic surface of potentials of the molecules M–S and M–O (M = Si, Ge, Sn) revealed that many of the B⋯M-S and B⋯M−O complexes should have a tetrel bond to M in which the axis of the M–S or M–O subunit should be approximately perpendicular to the axis of the nonbonding or π-bonding electron pair carried by B, a novel type of tetrel bond confirmed by geometry optimizations of the complexes.

## 1 Introduction

There has been considerable interest^1−6^ in characterizing the tetrel bond, especially because carbon is the prototype tetrel atom. Several noncovalent interactions have recently been the subject of IUPC definitions. So far these include the hydrogen bond,^7^ the halogen bond,^8^ the chalcogen bond^9^ and the pnictogen bond,^10^ while that of the tetrel bond is now in progress. Without prejudging the outcome for the last of these, if the coherent approach used in refs (7–10) is followed, a tetrel bond can be described as an attractive interaction between an electrophilic region associated with a tetrel atom in a molecular entity and a nucleophilic region of another, or the same, molecular entity. In this article, we investigate a range of pairwise interactions of simple, otherwise isolated, molecules. One molecule (the Lewis acid A) contains an electrophilic region associated with one of the tetrel atoms Si, Ge or Sn, while the other (the Lewis base B) carries a nucleophilic region composed of nonbonding (n) or π-bonding electron pairs. The electrophilic regions associated with tetrel atoms in molecules will be identified by means of the molecular electrostatic surface potentials (MESP), that is through the most positive region of the 0.001 e/bohr^3^ electron density iso-surface in the present case.

The tetrel-atom-containing molecules of interest here fall into two groups of Lewis acids A, namely

a H_3_M–X, where M = Si, Ge, or Sn and X = F, Cl, CN or H,
b M–O, M–S, where M = Si, Ge or Sn.

**Figure 1:**
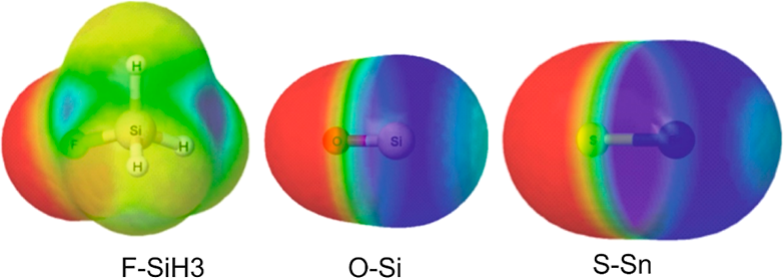
MESPs of H_3_SiF, O–Si and S–Sn on the 0.001 e/bohr^3^ electron density iso-surface, as calculated at the MP2/aug-cc-pVTZ level. The most electrophilic region is indicated by the darkest blue color. This is on the *C*_3_ axis of H_3_SiF, near to the Si atom; there are also three on the extensions of the Si–H bonds on the other side of the Si atom, but these are less electrophilic. Note that for O–Si and S–Sn the most electrophilic region is a band around the tetrel atom, with the cap at the Si and Sn atom less electrophilic.

Figure 1 shows the MESPs of H_3_SiF, Si–O and Sn–S calculated at the MP2/aug-cc-pVTZ level of theory. The dark blue regions are the most positive (electrophilic) while the red regions are the most negative (nucleophilic). It is clear from Figure 1 that the most electrophilic region of H_3_SiF lies on the *C*_3_ axis close to the Si atom, while that of SiO forms a belt around the Si atom perpendicular to the molecular axis but is less positive on the cap of Si. The corresponding MESPs of the other H_3_MX and MX defined in (a,b) are similar and are available in the Supporting Information (Figures S1–S2).

Lewis bases B discussed here as partners in forming tetrel bonds to the Lewis acids defined in (a) and (b) via n- or π-electron pairs are N_2_, OC, C_2_H_2_, PH_3_, C_2_H_4_, SC, HCN, HNC, H_2_O, PN and NH_3_. The aim is to determine the electrophilicities *E*_A_ of these Lewis acid molecules. This is possible using *abinitio* calculated equilibrium dissociation energy *D*_e_ for the process B⋯A = B + A, as set out in the next paragraph.

**Figure 2:**
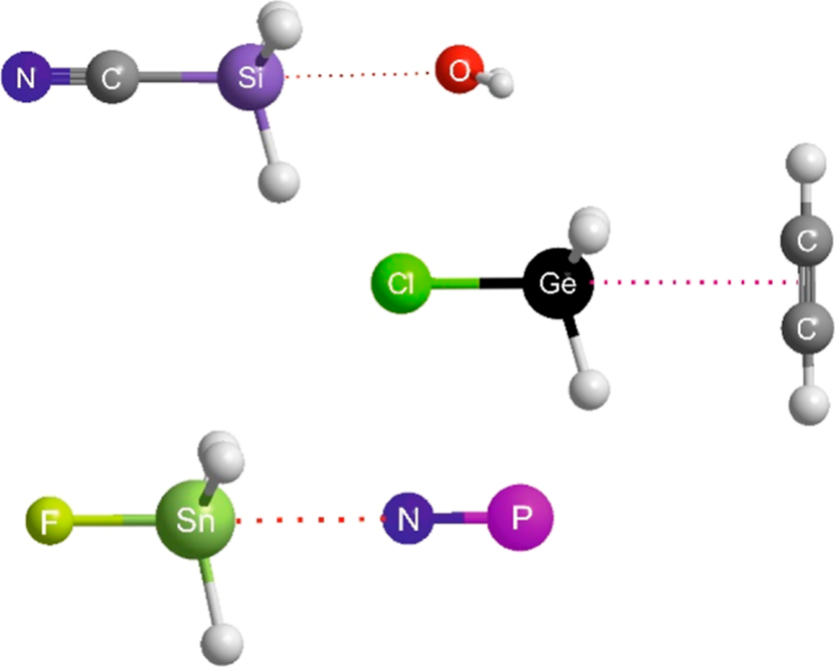
Optimized geometries at the CCSD(T)(F12c)/cc-pVDZ-F12 level for three of the B⋯H_3_MX complexes discussed here (B = H_2_O, HCCH, NP).

It was shown many years ago^11^ that the intermolecular stretching force constant *k*_σ_ of hydrogen-bonded complexes B⋯HX could be partitioned simply into a nucleophilicity assigned to the Lewis base B and an electrophilicity assigned to the Lewis acid HX. Subsequently, it was noted^12,13^ that *k*_σ_ is directly proportional to the equilibrium dissociation energy *D*_e_ of the complex. This evolved^14^ into eq 1
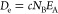
1which allows the definitions of the nucleophilicity *N*_B_ of the Lewis base B and the electrophilicity *E*_A_ of a general Lewis acid A that form complexes B⋯A. Sets of *N*_B_ and *E*_A_ values were determined^14^ from least-squares fits of *ab initio* calculated *D*_e_ values for selected complexes B⋯A. The constant *c* was set as 1.0 kJ mol^–1^ for convenience so that when *D*_e_ is available in kJ mol^–1^ the quantities *N*_B_ and *E*_A_ are dimensionless. For example, it was found that graphs of *D*_e_ versus *E*_HX_ for hydrogen-bonded complexes B⋯HX (X = F, Cl, Br, I, CN, CCH, CP) were straight lines through the origin when the Lewis base B was the same in each complex. The gradient of each graph then provides the nucleophilicity *N*_B_ of that base B. Correspondingly, graphs of *D*_e_ versus *N*_B_ for complexes B⋯A in which B was varied but the Lewis acid A was fixed yielded the electrophilicity *E*_A_ of the molecule A. In this article, we use the second approach to construct graphs of *D*_e_ versus *N*_B_ for complexes B⋯A in which the B are the 11 Lewis bases B listed in the title and A is one of the tetrel-atom containing molecules H_3_MX. The tetrel bonds in the B⋯H_3_MX complexes lie along the *C*_3_ axis of H_3_MX in each case, as seen for the examples of the optimized geometries of a selection of three complexes B···H_3_MX shown in Figure 2. The effect of the substitution of a single H atom in H_3_MH by F, Cl and CN on the electrophilicity of these Lewis acids, and hence on their propensity to form tetrel bonds, will thereby be established.

We also consider the diatomic molecules M–O, and M–S (M = Si, Ge or Sn) in complexes with the same set of Lewis bases B and examine the electrophilicities associated with the tetrel atoms M = Si, Ge and Sn in these molecules. We find that the tetrel bonds in most of the B···M–O/M–S complexes are of a different type from those involving the H_3_MX; with the axis of the n- or π electron pairs of molecules B approximately perpendicular to the M–O or M–S axis.

## 2 Computational Methods

The CCSD(T)-F12c computational level^15^ with the cc-pVDZ-F12 basis set^16^ has been employed to optimize the geometries of the monomers and complexes. The standard frozen-core approximation was used for all the complexes, except for those containing a Sn atom, for which 10 electrons have been correlated instead of the 4 used in the default calculations. This procedure avoids the mixing of the core and valence electrons in the complexes where Sn is present. The dissociation energies have been corrected for the basis set superposition error using Boys and Bernardi full counterpoise method.^18^ All these calculations have been carried out with the MOLPRO program.^17^

The MESP on the 0.001 e/bohr^3^ electron density iso-surface of the isolated Lewis acid monomers have been calculated at the MP2/aug-cc-pVTZ level with the Gaussian-16 program^19^ and analyzed with the Multiwfn program.^20^

## 3 Results

### 3.1 Tetrel Bonds in B⋯H_3_MX, M = Si, Ge, or Sn and X = H, F, Cl or CN

Figures 1 and S1 reveal that the most electrophilic region of each of the H_3_MX molecules lies on the *C*_3_ symmetry axis near to the tetrel atom. The optimized geometries of the B⋯H_3_MX when the Lewis bases B are N_2_, CO, C_2_H_2_, PH_3_, C_2_H_4_, NCH, CNH, CS, NP, H_2_O and NH_3_ reflect this, as may be seen from Figure 2 and the optimized Cartesian coordinates available in Table S1. Thus, when B is N_2_, CO, CS, NP, HCN or HNC the nonbonding electron pair on the C_∞_ axis of the diatomic or linear molecule interacts with the relatively positive region near the tetrel atom to give a complex of the type B⋯H_3_MX having *C*_3*v*_ symmetry. When B = NH_3_ or PH_3_, the axial n-pair of these molecules acts in the same way, while when B = H_2_O one of the two n-pairs on O fulfils this role. In the case of B = ethene or ethyne, the π electrons interact with the axial electrophilic region near the tetrel atom M so that the CC axis is perpendicular to the *C*_3_ axis of H_3_MX.

**Figure 3:**
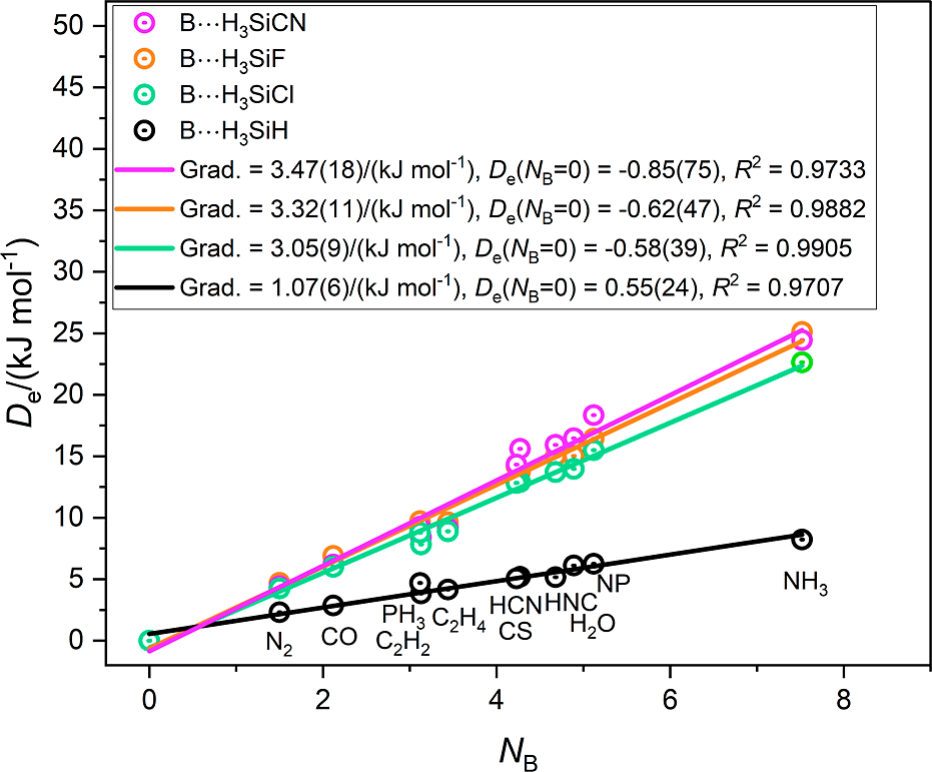
Graphs of *D*_e_ of the complexes B⋯H_3_SiX (X = CN, F, Cl, H) versus the nucleophilicities *N*_B_ of the Lewis bases B = N_2_, CO, PH_3_, C_2_H_2_, C_2_H_4_, HCN, HNC, CS, H_2_O, NP and NH_3_.

Figure 3 shows the *D*_e_ versus *N*_B_ graphs for the B⋯H_3_SiX complexes when X is either CN, F, Cl or H. Values of *N*_B_ used in Figure 3, and all similar Figures, are from ref (14), except for those of B = SC, HNC and PN which are from the gradients of *D*_e_ versus *E*_HX_ graphs that were published^21^ after ref (14). As expected from eq 1 each set of 12 points (including the origin) in Figure 3 yields a straight line through the origin. The gradients (and therefore the axial electrophilicities *E*_H3SiX_ of the H_3_SiX molecules) lie in the order X = CN ∼ F ∼ Cl ≫ H. Clearly, the H_3_SiH molecule forms very weak tetrel bonds, while those formed by H_3_SiX, (X = CN, Cl, F) are much stronger, and of nearly equal strength.

**Figure 4:**
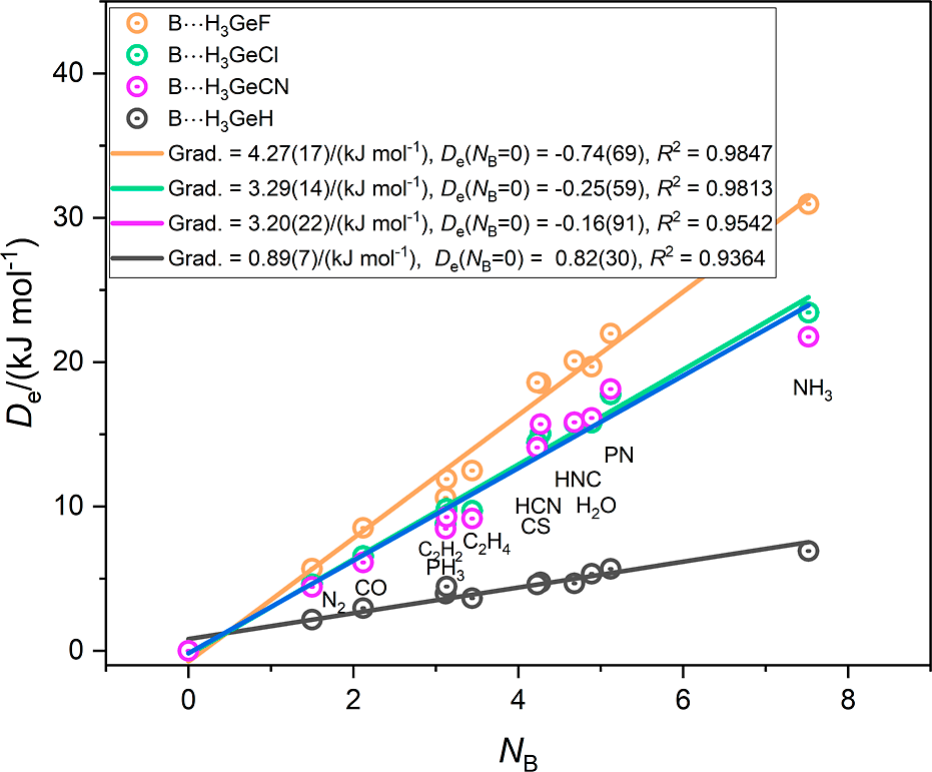
Graphs of *D*_e_ of the complexes B⋯H_3_GeX (X = CN, F, Cl, H) versus the nucleophilicities *N*_B_ of the Lewis bases B = N_2_, CO, PH_3_, C_2_H_2_, C_2_H_4_, HCN, HNC, CS, H_2_O, NP and NH_3._.

**Figure 5:**
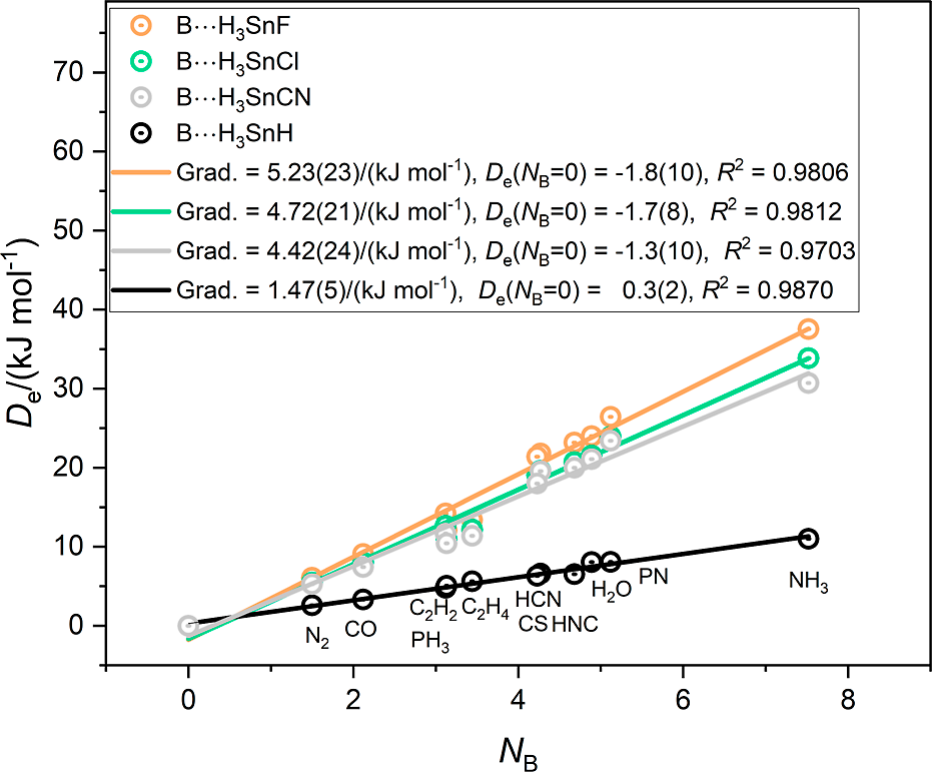
Graphs of *D*_e_ of the complexes B⋯H_3_SnX (X = CN, F, Cl, H) versus the nucleophilicities *N*_B_ of the Lewis bases B = N_2_, CO, PH_3_, C_2_H_2_, C_2_H_4_, HCN, HNC, CS, H_2_O, NP and NH_3_.

The graphs for the corresponding complexes involving the Group 14 atoms of rows 3 and 4 of the Periodic Table, namely B⋯H_3_GeX and B⋯H_3_SnX (X = CN, F, Cl, H), are in Figures 4 and 5, respectively. In Figure 4, the gradients (and therefore the electrophilicities of the H_3_GeX molecules) are in the order X = *F* > Cl ∼ CN ≫ H. Figure 5 shows that for the complexes involving H_3_SnX as the Lewis acids the order is again X = *F* > Cl ∼ CN ≫H. It is an interesting observation that the electrophilicities of H_3_GeX and H_3_SnX for X = CN are smaller than those of the corresponding molecules with X = F despite the fact that the CN group is more electron-withdrawing than F.

It was shown elsewhere^22^ that for hydrogen-bonded complexes B⋯HX (and other related types such as halogen-bonded complexes) it is possible to define a reduced electrophilicity Ξ_HX_. Examination of the MESPs of simple Lewis acids (such as the hydrogen halides) established that the value of the potential on the molecular axis near to H is the maximum (positive) potential σ_max_ on the defined iso-surface and is therefore the most electrophilic region of the molecule. Dividing eq 1 by σ_max_ gives
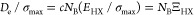
2where Ξ_HX_ = (*E*_HX_/σ_max_). As was shown in detail in ref (22), plots of *D*_e_/σ_max_ versus *N*_B_ in which B varies but HX is held constant are necessarily straight–line graphs but now of gradient Ξ_HX_ = *E*_HX_/σ_max_, in which Ξ_A_is dimensionless if *c* = 1.0 kJ mol^–1^ and *D*_e_ and σ_max_ are in kJ mol^–1^. In this section, we are examining Lewis acids H_3_MX (M = Si, Ge, Sn; X = F, Cl, CN, H), all of which have σ_max_ on the *C*_3_ symmetry axis near to the tetrel atom M. It is therefore of interest to discover whether Ξ_H3MX_ = (*E*_H3MX_/σ_max_) is an intrinsic property of the H_3_MX molecule, independent of the atom/group X attached to M.

**Figure 6:**
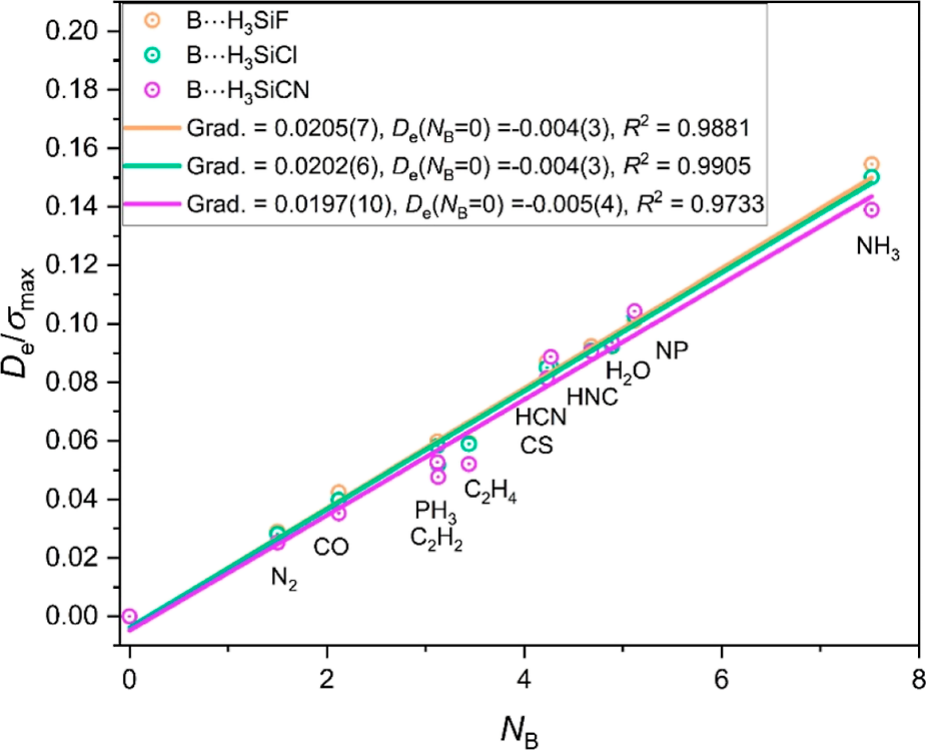
Graphs of *D*_*e*_/σ_max_ for the series of complexes B⋯H_3_SiX (X = F, Cl, CN) versus the nucleophilicities *N*_B_ of the Lewis bases B = N_2_, CO, PH_3_, C_2_H_2_, C_2_H_4_, HCN, HNC, CS, H_2_O, NP and NH_3_.

Figure 6 displays three graphs of *D*_e_/σ_max_ for the complexes B⋯H_3_SiX (X = F, Cl, CN) plotted against *N*_B_ for the series of Lewis bases B = N_2_, CO, PH_3_, C_2_H_2_, C_2_H_4_, HCN, HNC, CS, H_2_O, NP and NH_3_. Each straight line is a linear regression fit of the points for one of the three series B⋯H_3_SiX (X = F, Cl, CN). Note that the gradients of the three fitted straight lines in Figure 6, which according to eq 2 define the reduced electrophilicities Ξ_H3SiX_ of the H_3_SiX (X = F, Cl, CN) molecules, are identical within the standard errors of the fits. The corresponding graphs for the series B⋯H_3_GeX and B⋯H_3_SnX are available as Figures S3 and S4 of the Supporting Information. The three straight lines are again conflated within the standard errors of the fits in each of Figures S3 and S4.

The results for the reduced electrophilicities from Figures 6, S3 and S4 are set out in Table 1. The mean values are in the final column of the Table 1, from which it is concluded that the reduced electrophilicities of the tetrel atoms in the H_3_SiX and H_3_GeX are identical within the quoted errors, but that of Sn in the H_3_SnX is slightly larger. Reduced electrophilicities and reduced nucleophilicities can provide in suitable cases^23^ a method of obtaining *D*_e_ values for complexes without the need to carry out *ab initio* calculations.

**Table 1:** Reduced Electrophilicities of the Lewis Bases H_3_MX (M = Si, Ge, Sn; X = F, Cl, CN)

Lewis acid	Reduced electrophilicity Ξ_H3SiX_ (gradient of *D*_e_/σ_max_ versus *N*_B_ graph) for H_3_MX^a^			
	X = F	X = Cl	X = CN	mean value of Ξ_H3SiX_
H_3_SiX	0.0206(7)	0.0202(6)	0.0197(10)	0.0202(8)
H_3_GeX	0.0240(10)	0.0210(9)	0.0192(13)	0.0214(11)
H_3_SnX	0.0244(11)	0.0244(11)	0.0225(12)	0.0237(11)

a From Figures 6, S3 and S4.

The reason that X = H has been excluded from Figures 6, S3 and S4, and therefore from Table 1, is that the *D*_e_ values for the B⋯H_3_MH complexes are all very small (for a given complex around 3 or 4 times smaller than those having X = F, Cl or CN). For such small *D*_e_ values, other than electrostatic considerations (such as dispersion) are likely to have more significant effects. Certainly, the values of Ξ_H3SiX_ determined from graphs of *D*_e_/σ_max_ versus *N*_B_ for complexes B⋯H_3_MH are considerably smaller at 0.0130(20).

The conclusion from this section is that the series of molecules H_3_MX (M = Si, Ge, Sn; X = F, Cl, CN, H) form complexes with Lewis bases by means of tetrel bonds in which the nonbonding or π-bonding electron pairs of B interact with the electrophilic region of H_3_MX on the *C*_3_ axis near to the tetrel atom M.

### 3.2 Tetrel Bonds in which Lewis Bases B Interact with Electrophilic Regions in Tetrel Monoxides M–O and Monosulphides M–S (M = Si, Ge, Sn)

The MESPs of the tetrel monoxides Si–O, Ge–O and Sn–O and the tetrel monosulfides Si–S, Ge–S and Sn–S all possess regions that are highly positive, as can be seen in Figures 1 and S1. The positive (dark blue) regions are in all cases centered near to the tetrel atoms. However, the most positive regions (darkest blue) do not extend to the tip of the tetrel atom, but instead form a band around the atom (see Figures 1 and S1). This indicates that an essentially electrostatic interaction of the nonbonding electron pair of a Lewis base such as HCN will not yield a linear complex, for example S–Sn⋯NCH, and it is expected that the Lewis base should form an approximately right-angled geometry, with the axis of HCN perpendicular to that of S–Sn.

**Figure 7:**
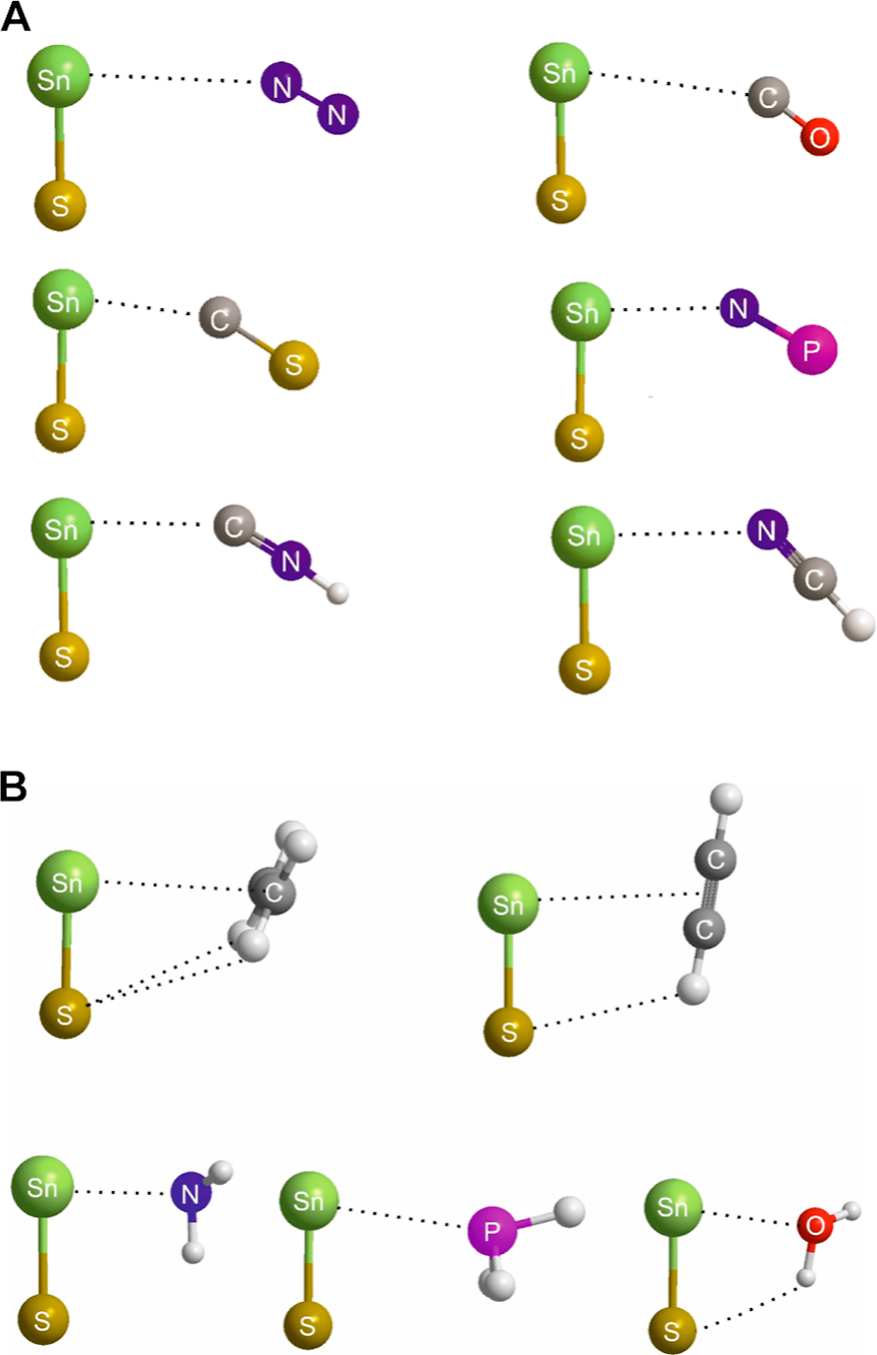
(A) Geometries of complexes formed between Sn–S and each of six diatomic or linear molecules acting as donors of nonbonding electron pairs to the electrophilic band that surrounds the tetrel atom Sn, as can be seen in the MESP of Sn–S shown in Figure S2. (B) Geometries of complexes formed between Sn–S and each of five Lewis bases C_2_H_4_, C_2_H_2_, NH_3_, PH_3_ and H_2_O.

Optimized geometries obtained from *ab initio* calculations at the CCS(D)T(F12c)/cc-pVDZ-F12 level, for the complexes B⋯Sn–S in which B is one of the diatomic/linear molecules N_2_, CO, CS, NCH, CNH, NP are set out in Figure 7A. The axis of each diatomic or linear molecule lies, at least approximately, perpendicular to the Sn–S molecule axis, with the leading atom of B pointing at the tetrel atom of the Lewis acid. The other end of B is sufficiently far from the nucleophilic S atom of Sn–S that any secondary interaction is not likely to be large. The geometries of five other complexes B⋯Sn–S, optimized at the same level of theory, are shown in Figure 7B. In these, the Lewis bases are NH_3_, H_2_O, PH_3_, C_2_H_2_ and C_2_H_4_. Some Lewis bases, such as NH_3_ and H_2_O, are more strongly bound to Sn–S than those in Figure 7A, leading to shorter intermolecular distances, and hence greater opportunities for secondary interactions. The Lewis bases having π-bonds (ethyne and ethene) also exhibit obvious secondary H-bond interactions, as indicated.

A similar range of geometries occurs when the Lewis acid is Ge–S, as shown in Figure S5. The M–S bond is longer for M = Ge and Sn, and therefore the secondary interactions of H of the Lewis bases B with the nucleophilic S atoms of SnS (Figure 7B) and GeS (Figure S2) should be small, but secondary interactions appear to be of increasing importance in the M-O complexes and in Si–S complexes, in both of which Lewis acids the internuclear distance is shorter. Nevertheless, Figures 7A,B and S5 show that there is an interesting type of tetrel bond formed by the Sn and Ge atoms of Sn–S and Ge–S respectively with the nucleophilic regions (nonbonding or π-bonding electron pairs) of the Lewis bases B. The tetrel bond is approximately perpendicular to the axes of the SnS and GeS submolecules, as predicted from the electrophilic band that surrounds the tetrel atoms in the MESPs of the M–O and M–S (see Figures 1 and S1).

**Figure 8:**
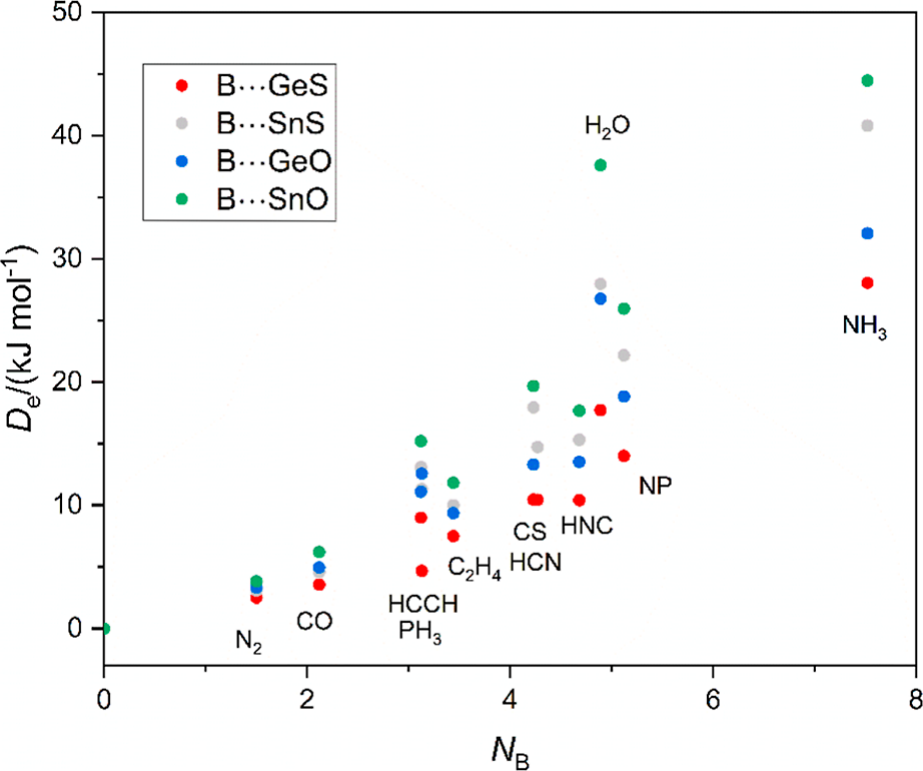
*D*_e_ versus *N*_B_ for all tetrel-bonded complexes of the type B⋯Ge–O, B⋯Ge–S, B⋯Sn–O, and B⋯Sn–S in which B = N_2_, CO, PH_3_, C_2_H_2_, C_2_H_4_, HCN, HNC, CS, H_2_O, NP, and NH_3_. Equation 1 is clearly not applicable to all points associated with a given B⋯M-O or B⋯M-S series of complexes because a number of points deviate significantly from collinearity, presumably as a result of secondary interaction, notably for B = H_2_O and NH_3_.

Even when complexes of the type B⋯Si–O and B⋯Si–S are ignored, the fact that the *D*_e_ versus *N*_B_ graphs cannot be fitted completely by means of eq 1 is illustrated by Figure 8, in which all relevant points associated with the tetrel-bonded complexes B⋯Ge–O, B⋯Ge–S, B⋯Sn–O, and B⋯Sn–S are displayed as a scatter diagram, with no attempt to fit each set to eq 1, which would be inappropriate.

**Figure 9:**
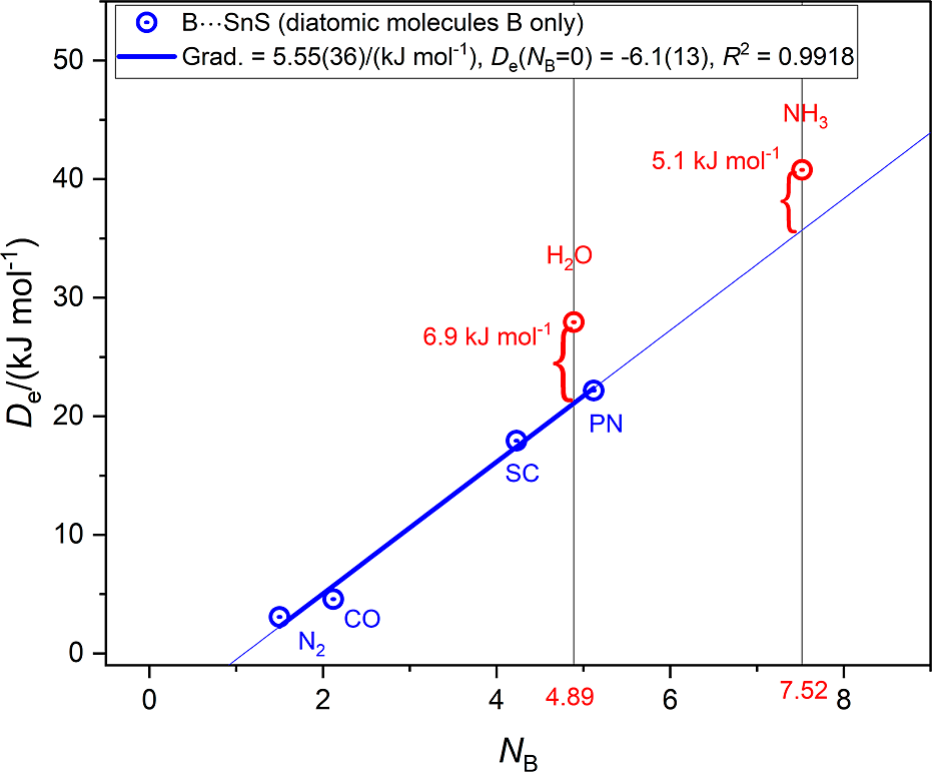
*D*_e_ versus *N*_B_ for those B⋯SnS complexes having B = N_2_, CO, CS and NP and which are fitted by linear regression. The fitted line is extrapolated to *N*_B_ = 9. The points for B = H_2_O and NH_3_ are shown but were not included in the fit. The distance of these points from the intersections of the *N*_B_ values (4.89 and 7.52, respectively) of H_2_O and NH_3_ with the extrapolated line then gives a rough indication of the secondary hydrogen-bond interaction strength in H_2_O⋯SnS and H_3_N⋯SnS.

To provide a rough estimate of the extent of the secondary hydrogen-bond interactions in complexes that lead to the large deviations from eq 1, as in the case of B = H_2_O and NH_3_, we consider the example of the B···SnS complexes. We first note from Figure 7A that the complexes least likely to engage in secondary interactions are those involving the diatomic Lewis bases N_2_, CO, CS and NP because these are of relatively low polarity, unlike HCN and HNC, which moreover have a terminal H atom that could be involved in a hydrogen bond to S of Sn–S. Figure 9 displays a graph *D*_e_ versus *N*_B_ for the four complexes SSn⋯N_2_, SSn⋯CO, SSn⋯CS, and SSn⋯NP. These four points were fitted by linear regression to give a reasonable straight line having *R*^2^ = 0.9918. The points for SSn⋯H_2_O and SSn⋯NH_3_ were then added but not included in the linear regression. The deviations of the observed *D*_e_ of the SSn⋯H_2_O and SSn⋯NH_3_ points from the regression line are indicated by the red braces and have values 6.9 and 5.1 kJ mol^–1^, respectively.

The values seem to have a reasonable magnitude for a secondary hydrogen bond but it should be emphasized that the analysis presented is intended only to illustrate that secondary interactions are present and should not be taken to have quantitative significance. It is worth noting, however, that Klemperer and co-workers^24^ considered that NH_3_ is reluctant to participate as a hydrogen donor in hydrogen-bond formation. The larger value for B = H_2_O is not inconsistent with this conclusion.

## 4 Conclusions

The equilibrium dissociation energies *D*_e_ of two types of tetrel-bonded complexes were calculated at the CCSD(T)(F12c)/cc-pVDZ-F12 level of theory. The first type of complex consisted of those in which a molecule of the general type H_3_MX (M = Si, Ge or Sn and X = F, Cl, CN or H) acts as the Lewis acid and interacts with one of the electron-pair donor molecules B = N_2_, OC, C_2_H_2_, PH_3_, C_2_H_4_, SC, HCN, HNC, H_2_O, PN and NH_3_ acting as the Lewis base. The MESPs of the H_3_MX molecules indicate that the most electrophilic region lies on the *C*_3_ axis, near to the tetrel atom in all examples of M and X considered, and that a lower value is associated with the electrophilic region opposite each H atom. The calculated geometries of the B⋯H_3_MX complexes confirm this conclusion about electrophilic regions, with the axis of a nonbonding or π-bonding electron pair of B lying along the *C*_3_ axis of the Lewis acid. Graphs of *D*_e_ versus the nucleophilicity *N*_B_ of B establish that the order of the electrophilicity *E*_H_3_MX_ of the Lewis acids H_3_MX is X = CN ∼ F ∼ Cl ≫ H for H_3_SiX, and X = F > Cl ∼ CN ≫ H for both H_3_GeX and H_3_SnX. Division of *D*_e_ by the maximum value σ_max_ of the MESP of the H_3_MX molecule for each series B⋯H_3_MX established that there exists a reduced electrophilicity which is identical for H_3_Si X and H_3_GeX and only slightly larger for the H_3_SnX.

The other group of tetrel bonds considered were those B⋯M-O/S (namely, involving tertel monoxides or monosufides) formed by the series of Lewis bases B defined in the preceding paragraph interacting with the tetrel-chalcogen diatomic molecules M-O/S, in which M = Si, Ge or Sn. Examination of the MESPs of the various M–O/S molecules revealed that the most electrophilic region of each of these M–O/S molecules lies in a band around the tetrel atom and that there is a cap of lower electrophilicity at the M end of the M–O/S molecule. This suggested that a nonbonding or π-bonding electron pair associated with the Lewis base B should interact with the tetrel atom M so that the axis of the electron pair of B (n- or π-type) lies approximately perpendicular to the M–O/S axis. Geometry optimizations at the CCSD(T)(F12c)/cc-pVDZ-F12 level established that this novel type of tetrel bond is present in most (but not all) of the 44 complexes B⋯M-O/S investigated. Attempts to determine the electrophilicities of the various tetrel-chalcogen diatomic molecules were hampered by the presence of weak secondary interactions formed with the nucleophilic O or S atom of M-O/S and, for example, one or more H atoms of the molecule B.

## Supporting Information Available

Tables S1–S6: optimized Cartesian coordinates calculated at the CCSD(T)(F12c)/cc-pVDZ-F12 level of theory for all complexes investigated. Figures S1 and S2: MESPs on the 0.001 e/bohr^3^ iso-surface calculated at the MP2/aug-cc-pVTZ level of theory for various H_3_MX, M–O and M–S molecules (M = Si, Ge, Sn; X = H, F, Cl, CN). Figures S3–S6: Chem-3D models of various optimized B⋯GeO and B⋯GeS geometries (PDF)
