# Supplementary material for: Ab Initio Investigation of Tetrel Bonds in Isolated Complexes Formed Between a Lewis Acid H3MX, M–O or M–S (M = Si, Ge, or Sn) and the Lewis Bases B = N2, CO, HCCH, PH3, C2H4, HCN, CS, HNC, NP, H2O, and NH3
Source: J Phys Chem A. 2024 Jul 15;128(29):5963–8. doi: 10.1021/acs.jpca.4c03438 (PMC11284776; doi:10.1021/acs.jpca.4c03438)
Supplement: Supplementary file 1 — jp4c03438_si_001.pdf [file jp4c03438_si_001.pdf]

SUPPLEMENTARY MATERIAL

An ab initio investigation of tetrel bonds in isolated complexes formed between a Lewis acid H_3MX, M-O or M-S (M = Si, Ge, or Sn) and the Lewis bases B = N_2, CO,HCCH, PH_3, C_2H_4, HCN, CS, HNC, NP, H_2O, andNH_3

Ibon Alkorta[a] and Anthony C. Legon[b]
[a]Instituto de Química Médica (IQM-CSIC), Juan de la Cierva, 3, E-28006 Madrid, Spain,
[b] School of Chemistry, University of Bristol, Cantock's Close, Bristol BS8 1TS, U. K

Index

Pg. S2-S10 Table S1. Optimised geometries (Å) and energies (au) of $XSiH_3\cdots B$ complexes calculated at the CCSD(T)(F12c)/cc-pVDZ-F12 level. X= H, F, Cl and CN; B= N_2, CO, HCCH, PH_3, HCN, H_2CCH_2, HNC, H_2O, NH_3, CS, NP

Pg. S11-S19 Table S2. Optimised geometries (Å) and energies (au) of $XGeH_3\cdots B$ complexes calculated at the CCSD(T)(F12c)/cc-pVDZ-F12 level. X= H, F, Cl and CN; B= N_2, CO, HCCH, PH_3, HCN, H_2CCH_2, HNC, H_2O, NH_3, CS, NP

Pg. S20-S28 Table S3. Optimised geometries (Å) and energies (au) of $XSnH_3\cdots B$ complexes calculated at the CCSD(T)(F12c)/cc-pVDZ-F12 level. X= H, F, Cl and CN; B= N_2, CO, HCCH, PH_3, HCN, H_2CCH_2, HNC, H_2O, NH_3, CS, NP

Pg. S29-S31 Table S4. Optimised geometries (Å) and energies (au) of $ZSi\cdots B$ complexes calculated at the CCSD(T)(F12c)/cc-pVDZ-F12 level. Z= O and S; B= N_2, CO, HCCH, PH_3, HCN, H_2CCH_2, HNC, H_2O, NH_3, CS, NP

Pg. S32-S34 Table S5. Optimised geometries (Å) and energies (au) of $ZGe\cdots B$ complexes calculated at the CCSD(T)(F12c)/cc-pVDZ-F12 level. Z= O and S; B= N_2, CO, HCCH, PH_3, HCN, H_2CCH_2, HNC, H_2O, NH_3, CS, NP

Pg. S35-S37 Table S6. Optimised geometries (Å) and energies (au) of $ZSn\cdots B$ complexes calculated at the CCSD(T)(F12c)/cc-pVDZ-F12 level. Z= O and S; B= N_2, CO, HCCH, PH_3, HCN, H_2CCH_2, HNC, H_2O, NH_3, CS, NP

Pg. S38 Figure S1. Molecular electrostatic surface potentials on the 0.001 e/bohr^3 iso-surfaces of H_3SiH, H_3SiCl and H_3SiCN calculated at the MP/aug-cc-pVTZ level of theory.

Pg. S39 Figure S2. Molecular electrostatic surface potentials on the 0.001 e/bohr^3 iso-surfaces of SiS, GeO, GeS, SnO and SnS calculated at the MP/aug-cc-pVTZ level of theory

Pg. S40 Figures S3 and S4. Graphs of D_e/σ_{max} versus N_B for the series of complexes $H_3GeX\cdots B$ and $H_3SnX\cdots B$, respectively, for X = F, Cl, CN and B = N_2, CO, HCCH, PH_3, C_2H_4, HCN, CS, HNC, NP, H_2O, andNH_3.

Pg. S41 Figure S5. Geometries of $B\cdots GeO$ complexes optimized at the CCSD(T)(F12c)/cc-pVDZ-F12 level

Pg. S41 Figure S6. Geometries of $B\cdots GeS$ complexes optimized at the CCSD(T)(F12c)/cc-pVDZ-F12 level

Table S1. Optimised geometries (Å) and energies (au) of XSiH3···B complexes calculated at the CCSD(T)(F12c)/cc-pVDZ-F12 level. X= H, F, Cl and CN; B= N2, CO, HCCH, PH3, HCN, H2CCH2, HNC, H2O, NH3, CS, NP

HSiH3···B, B= N2, CO, HCCH, PH3, HCN, H2CCH2, HNC, H2O, NH3, CS, NP

$HSiH_3\cdots N_2$	CCSD(T)-F12C/CC-PVDZ-F12 ENERGY=-400.85457845<br>N 0.0000000000 0.0000729069 -4.3743999543<br>N 0.0000000000 -0.0000293655 -3.2753674636<br>H 0.0000000000 1.3944531844 -0.1919116368<br>H -1.2077360495 -0.6972071680 -0.1916407031<br>H 1.2077360495 -0.6972071680 -0.1916407031<br>Si 0.0000000000 -0.0000099921 0.2980190305<br>H 0.0000000000 -0.0000723955 1.7769414304
$HSiH_3\cdots CO$	CCSD(T)-F12C/CC-PVDZ-F12 ENERGY=-404.63722781<br>O 0.0000000000 0.0000425561 -4.4519340309<br>C 0.0000000000 -0.0000059854 -3.3217075267<br>H 0.0000000000 1.3951443206 -0.1660969270<br>H -1.2083186416 -0.6976052751 -0.1661070600<br>H 1.2083186416 -0.6976052751 -0.1661070600<br>Si 0.0000000000 -0.0000827929 0.3212533333<br>H 0.0000000000 0.0001124540 1.8006992713
$HSiH_3\cdots HCCH$	CCSD(T)-F12C/CC-PVDZ-F12 ENERGY=-368.65812170<br>C 0.0000000000 -0.5943304331 -2.9968892065<br>C 0.0000000000 0.6111521301 -2.9945345969<br>H 0.0000000000 -1.6576462455 -2.9988479761<br>H 0.0000000000 1.6744500774 -2.9905551406<br>H 0.0000000000 1.3831691970 0.1980337930<br>H -1.2099593504 -0.7092709348 0.2213448916<br>H 1.2099593504 -0.7092709348 0.2213448916<br>Si 0.0000000000 -0.0078299069 0.6981635751<br>H 0.0000000000 0.0095375967 2.1780496641
$HSiH_3\cdots PH_3$	CCSD(T)-F12C/CC-PVDZ-F12 ENERGY=-634.15500363<br>P 0.0000991994 -0.0000446673 -3.5513172253<br>H 1.1898127688 0.0610516087 -4.3120746858<br>H -0.5420093524 -1.0610581280 -4.3117267095<br>H -0.6479048104 1.0000010178 -4.3115202908<br>H 0.0522508103 1.3955076831 -0.1111806499<br>H -1.2343808752 -0.6526448534 -0.1115086662<br>H 1.1822927574 -0.7430013556 -0.1112743923<br>Si 0.0000000473 0.0000311261 0.3721940045<br>H -0.0001605452 0.0001644081 1.8526230617
$HSiH_3\cdots NCH$	CCSD(T)-F12C/CC-PVDZ-F12 ENERGY=-384.75127796<br>H 0.0000000000 -0.0000370267 -5.1149998802<br>C 0.0000000000 -0.0000418735 -4.0481401460<br>N 0.0000000000 -0.0000465528 -2.8934057768<br>H 0.0000000000 1.3993049179 -0.0233124179<br>H -1.2115285518 -0.6996443590 -0.0237596267

	H 1.2115285518 -0.6996443590 -0.0237596267<br>Si 0.0000000000 0.0000579131 0.4494100597<br>H 0.0000000000 0.0000581795 1.9316170179
$HSiH_3\cdots H_2CCH_2$	CCSD(T)-F12C/CC-PVDZ-F12 ENERGY=-369.91027620<br>C 0.0369984173 -0.6667900350 -3.4423287436<br>C 0.0369912687 0.6667649501 -3.4423296514<br>H 0.9066828447 -1.2315085729 -3.7526891258<br>H -0.8328482233 -1.2307341304 -3.1303993062<br>H -0.8328624298 1.2307000155 -3.1304031487<br>H 0.9066705272 1.2314921388 -3.7526884965<br>Si -0.0295296632 0.0000159963 0.2362362738<br>H 0.7023619340 -1.2097025398 -0.1934022264<br>H -1.3831139825 -0.0000391199 -0.3586658695<br>H 0.7024785376 1.2097671415 -0.1931124850<br>H -0.1496614700 0.0000365263 1.7113800655
$HSiH_3\cdots OH_2$	CCSD(T)-F12C/CC-PVDZ-F12 ENERGY=-367.81148675<br>O 0.0000000000 0.0177334985 -2.6781391536<br>H 0.7585756423 -0.3576454104 -3.1282405547<br>H -0.7585756423 -0.3576454104 -3.1282405547<br>H 0.0000000000 1.4173029805 0.0217336732<br>H -1.2120914440 -0.6851406563 0.0173641067<br>H 1.2120914440 -0.6851406563 0.0173641067<br>Si 0.0000000000 0.0169847848 0.4872382185<br>H 0.0000000000 0.0078543528 1.9695451512
$HSiH_3\cdots NH_3$	CCSD(T)-F12C/CC-PVDZ-F12 ENERGY=-347.94501045<br>N -0.0000542935 -0.0000441328 -2.5627691102<br>H 0.9168246848 0.1948830774 -2.9453618381<br>H -0.2897859076 -0.8912974397 -2.9458528421<br>H -0.6271609102 0.6967695404 -2.9452648065<br>H 0.1274028932 1.3987909982 0.1636393902<br>H -1.2751784519 -0.5890524625 0.1640513242<br>H 1.1478647162 -0.8103107365 0.1641634882<br>Si 0.0002088892 -0.0000934720 0.6184869598<br>H -0.0001216203 0.0003614669 2.1033031657
$HSiH_3\cdots CNH$	CCSD(T)-F12C/CC-PVDZ-F12 ENERGY=-384.72750744<br>H 0.0000000000 0.0005426267 -5.1584329021<br>N 0.0000000000 0.0005110185 -4.1615093011<br>C 0.0000000000 0.0004717559 -2.9913246920<br>H 0.0000000000 1.3978707262 0.0240018053<br>H -1.2122025219 -0.6996252618 0.0286215185<br>H 1.2122025219 -0.6996252618 0.0286215185<br>Si 0.0000000000 -0.0001736524 0.5006643759<br>H 0.0000000000 0.0000348883 1.9830072803
$HSiH_3\cdots NP$	CCSD(T)-F12C/CC-PVDZ-F12 ENERGY=-687.02490440<br>P 0.0000000000 -0.0006246638 -4.5156463897<br>N 0.0000000000 0.0004889712 -3.0234898812<br>H 0.0000000000 1.4003696729 -0.2061274157<br>H -1.2126916540 -0.6999570542 -0.2066861945<br>H 1.2126916540 -0.6999570542 -0.2066861945<br>Si 0.0000000000 -0.0000286883 0.2628588535

	H 0.0000000000 -0.0002911816 1.7457772219
$HSiH_3\cdots CS$	CCSD(T)-F12C/CC-PVDZ-F12 ENERGY=-727.16661942<br>S 0.0000000000 0.0000414185 -4.7400855802<br>C 0.0000000000 -0.0002313527 -3.2033474052<br>H 0.0000000000 1.3984394994 -0.1683122963<br>H -1.2107443929 -0.6990948017 -0.1684031853<br>H 1.2107443929 -0.6990948017 -0.1684031853<br>Si 0.0000000000 0.0001937886 0.3084422513<br>H 0.0000000000 -0.0002537483 1.7901094009

FSiH3···B, B= N2, CO, HCCH, PH3, HCN, H2CCH2, HNC, H2O, NH3, CS, NP

$FSiH_3\cdots N_2$	CCSD(T)-F12C/CC-PVDZ-F12 ENERGY=-500.09721600<br>N 0.0000000000 0.0000000000 -4.6925277779<br>N -0.0000000000 0.0000000000 -3.5937783539<br>Si 0.0000000000 0.0000000000 -0.3715532235<br>H 0.7005827797 -1.2134449694 -0.8278730835<br>H -1.4011655595 0.0000000000 -0.8278730835<br>H 0.7005827797 1.2134449694 -0.8278730835<br>F 0.0000000000 0.0000000000 1.2258701152
$FSiH_3\cdots CO$	CCSD(T)-F12C/CC-PVDZ-F12 ENERGY=-503.88048972<br>O 0.0000000000 0.0000000000 -4.7129458619<br>C -0.0000000000 0.0000000000 -3.5835974801<br>Si 0.0000000000 0.0000000000 -0.3734398567<br>H 0.7014908126 -1.2150177285 -0.8236455119<br>H -1.4029816252 0.0000000000 -0.8236455119<br>H 0.7014908126 1.2150177285 -0.8236455119<br>F 0.0000000000 0.0000000000 1.2253112437
$FSiH_3\cdots HCCH$	CCSD(T)-F12C/CC-PVDZ-F12 ENERGY=-467.90149752<br>C 0.0065520339 -0.6029343237 -3.2332397237<br>C 0.0065456953 0.6029529948 -3.2332237859<br>H 0.0073512847 -1.6665142256 -3.2392307619<br>H 0.0073332476 1.6665330720 -3.2391857052<br>Si-0.0073795542 -0.0000377410 0.1449555913<br>H 0.6891192286 -1.2150569759 -0.3135134092<br>H -1.4169747554 0.0000579423 -0.2816099958<br>H 0.6890533091 1.2150161588 -0.3135162193<br>F 0.0183995106 -0.0000197870 1.7444581897
$FSiH_3\cdots PH_3$	CCSD(T)-F12C/CC-PVDZ-F12 ENERGY=-733.39905957<br>P -0.0000739269 0.0000526289 -3.7303326352<br>H 1.1964079984 -0.0234395080 -4.4798447967<br>H -0.6186460364 -1.0243116484 -4.4799274335<br>H -0.5778619112 1.0477656093 -4.4802074673<br>Si -0.0000449332 0.0000338710 -0.2947155461<br>H 0.7637365440 -1.1804011704 -0.7350699690<br>H -1.4042705053 -0.0712556532 -0.7348909291<br>H 0.6406497487 1.2515613964 -0.7349516641<br>F 0.0001008272 -0.0000556943 1.3067273724

$FSiH_3$···NCH	CCSD(T)-F12C/CC-PVDZ-F12 ENERGY=-483.99627417<br>H -0.0000000000 0.0000000000 -5.6103902774<br>C -0.0000000000 0.0000000000 -4.5433179152<br>N 0.0000000000 0.0000000000 -3.3895689185<br>Si -0.0000000000 0.0000000000 -0.4521026217<br>H 0.7046274829 -1.2204506008 -0.8777344786<br>H -1.4092549658 0.0000000000 -0.8777344786<br>H 0.7046274829 1.2204506008 -0.8777344786<br>F -0.0000000000 0.0000000000 1.1529746778
$FSiH_3$··H_2CCH_2	CCSD(T)-F12C/CC-PVDZ-F12 ENERGY=-469.15389169<br>C 0.0232691058 -0.6672122990 -3.2681953176<br>C 0.0232703244 0.6672144055 -3.2681964303<br>H 0.9364512626 -1.2316713811 -3.4043984807<br>H -0.8910739737 -1.2313975299 -3.1362996653<br>H -0.8910722567 1.2314015213 -3.1363053748<br>H 0.9364540411 1.2316716169 -3.4043969169<br>Si -0.0249515122 0.0000014281 0.0647201338<br>H 0.6935641850 -1.2158966917 -0.3554569648<br>H -1.4110638220 -0.0000070125 -0.4356280220<br>H 0.6935662567 1.2158997845 -0.3554526429<br>F -0.0884136110 -0.0000021081 1.6632701035
$FSiH_3$···OH_2	CCSD(T)-F12C/CC-PVDZ-F12 ENERGY=-467.05665879<br>O -0.1002024009 -0.0063730323 -2.9909357999<br>H 0.7412491827 -0.0122053368 -3.4508165440<br>H -0.6184434085 -0.6931244277 -3.4144455884<br>Si -0.0071438992 0.0026271108 -0.2030746562<br>H 0.5746977463 -1.3003418975 -0.5704899229<br>H -1.4206457336 0.1106828562 -0.5985375866<br>H 0.8064733362 1.1306085955 -0.6836721982<br>F 0.0240151768 0.0705688094 1.4007778758
$FSiH_3$···NH_3	CCSD(T)-F12C/CC-PVDZ-F12 ENERGY=-447.19335705<br>N -0.0000243581 0.0001028235 -2.8259894580<br>H 0.9383163587 0.0586463658 -3.2025845160<br>H -0.4185116357 -0.8417575555 -3.2027336190<br>H -0.5198625683 0.7835319319 -3.2025881202<br>Si -0.0000239205 -0.0000530431 -0.2884411232<br>H 0.7932984451 -1.1942128664 -0.6276412423<br>H -1.4309094819 -0.0899476643 -0.6275806637<br>H 0.6375135860 1.2840624679 -0.6276796795<br>F 0.0000271483 -0.0000614144 1.3294156679
$FSiH_3$···CNH	CCSD(T)-F12C/CC-PVDZ-F12 ENERGY=-483.97301420<br>H 0.0000000000 0.0000000000 -5.5871093447<br>N -0.0000000000 0.0000000000 -4.5900390351<br>C -0.0000000000 0.0000000000 -3.4218859536<br>Si 0.0000000000 0.0000000000 -0.4450780354<br>H 0.7056602019 -1.2222393227 -0.8642424502<br>H -1.4113204038 0.0000000000 -0.8642424502<br>H 0.7056602019 1.2222393227 -0.8642424502<br>F 0.0000000000 0.0000000000 1.1612312287
$FSiH_3$···NP	CCSD(T)-F12C/CC-PVDZ-F12 ENERGY=-786.27056244<br>P 0.0000000000 0.0000000000 -4.7210785423

	N -0.0000000000 0.0000000000 -3.2309326096<br>Si 0.0000000000 0.0000000000 -0.3862542849<br>H 0.7064272869 -1.2235679528 -0.7995000028<br>H -1.4128545738 0.0000000000 -0.7995000028<br>H 0.7064272869 1.2235679528 -0.7995000028<br>F -0.0000000000 0.0000000000 1.2211569544
$FSiH_3$···CS	CCSD(T)-F12C/CC-PVDZ-F12 ENERGY=-826.41187065<br>S -0.0000000000 0.0000000000 -4.8359727153<br>C 0.0000000000 0.0000000000 -3.3029840834<br>Si -0.0000000000 0.0000000000 -0.3435460081<br>H 0.7055513842 -1.2220508448 -0.7649523312<br>H -1.4111027684 0.0000000000 -0.7649523312<br>H 0.7055513842 1.2220508448 -0.7649523312<br>F 0.0000000000 0.0000000000 1.2617513097

ClSiH3···B, B= N2, CO, HCCH, PH3, HCN, H2CCH2, HNC, H2O, NH3, CS, NP

$ClSiH_3$···N_2	CCSD(T)-F12C/CC-PVDZ-F12 ENERGY=-860.06550356<br>Si -0.0000000000 0.0000000000 0.3864899414<br>Cl -0.0000000000 0.0000000000 2.4423118580<br>H 1.3990811591 0.0000000000 -0.0726275709<br>H -0.6995405796 -1.2116398258 -0.0726275709<br>H -0.6995405796 1.2116398258 -0.0726275709<br>N 0.0000000000 0.0000000000 -2.9211383354<br>N 0.0000000000 0.0000000000 -4.0199993237
$ClSiH_3$···CO	CCSD(T)-F12C/CC-PVDZ-F12 ENERGY=-863.84861862<br>Si -0.0000000000 0.0000000000 0.4074824609<br>Cl -0.0000000000 0.0000000000 2.4657810869<br>H 1.4009023989 0.0000000000 -0.0453737556<br>H -0.7004511994 -1.2132170656 -0.0453737556<br>H -0.7004511994 1.2132170656 -0.0453737556<br>C 0.0000000000 0.0000000000 -2.8794616465<br>O 0.0000000000 0.0000000000 -4.0089796882
$ClSiH_3$···HCCH	CCSD(T)-F12C/CC-PVDZ-F12 ENERGY=-827.86974858<br>Si 0.0139209200 0.8248038568 0.0000000000<br>Cl 0.0432026527 2.8845048441 0.0000000000<br>H 0.7067028997 0.3655203642 1.2145408330<br>H 0.7067028997 0.3655203642 -1.2145408330<br>H -1.3949748644 0.3968632484 0.0000000000<br>C 0.5304815905 -2.5918400592 0.0000000000<br>C -0.6753374874 -2.5823857755 0.0000000000<br>H 1.5940947157 -2.6072285770 0.0000000000<br>H -1.7389883201 -2.5790578588 0.0000000000
$ClSiH_3$···PH_3	CCSD(T)-F12C/CC-PVDZ-F12 ENERGY=-1093.36713193<br>Si 0.0000000000 0.0000000000 0.5092857437<br>Cl 0.0000000000 0.0000000000 2.5717249445<br>H -1.4032037534 0.0000000000 0.0650128244<br>H 0.7016018767 1.2152100971 0.0650128244<br>H 0.7016018767 -1.2152100971 0.0650128244<br>P -0.0000000000 0.0000000000 -3.0414742017<br>H 1.1956869648 0.0000000000 -3.7930843502

	H -0.5978434824 -1.0354952865 -3.7930843502<br>H -0.5978434824 1.0354952865 -3.7930843502
$ClSiH_3$···NCH	CCSD(T)-F12C/CC-PVDZ-F12 ENERGY=-843.96443722<br>Si -0.0000000000 0.0000000000 0.2672930763<br>Cl -0.0000000000 0.0000000000 2.3369574131<br>H 1.4073299620 0.0000000000 -0.1585432169<br>H -0.7036649810 -1.2187834986 -0.1585432169<br>H -0.7036649810 1.2187834986 -0.1585432169<br>N 0.0000000000 0.0000000000 -2.7300663960<br>C 0.0000000000 0.0000000000 -3.8839523764<br>H 0.0000000000 0.0000000000 -4.9511198404
$ClSiH_3$··H_2CCH_2	CCSD(T)-F12C/CC-PVDZ-F12 ENERGY=-829.12209175<br>Si 0.0174155967 0.9688144818 0.0000000000<br>Cl -0.0099493420 3.0292253917 0.0000000000<br>H -1.3793281541 0.5034579717 0.0000000000<br>H 0.7239951187 0.5284689361 1.2140055916<br>C -0.0207491281 -2.4224543878 0.6671504319<br>C -0.0207491281 -2.4224543878 -0.6671504319<br>H -0.9319553958 -2.2701382432 1.2313700985<br>H 0.8891717625 -2.5794486399 1.2316217942<br>H 0.8891717625 -2.5794486399 -1.2316217942<br>H -0.9319553958 -2.2701382432 -1.2313700985<br>H 0.7239951187 0.5284689361 -1.2140055916
$ClSiH_3$···OH_2	CCSD(T)-F12C/CC-PVDZ-F12 ENERGY=-827.02473053<br>Si -0.0115625765 0.0200269699 0.1772321313<br>Cl 0.0459140239 -0.0795254220 2.2452020559<br>H 1.3858622960 0.0463497721 -0.2774541857<br>H -0.7330712281 -1.1770170684 -0.2774541857<br>H -0.7278986428 1.2607574321 -0.1560409964<br>O -0.0299009934 0.0517900398 -2.6544583468<br>H -0.8591184038 -0.0325319415 -3.1286203378<br>H 0.4577326897 0.7277523918 -3.1286203378
$ClSiH_3$···NH_3	CCSD(T)-F12C/CC-PVDZ-F12 ENERGY=-807.16090081<br>Si 0.0000000000 0.0000000000 -0.3355115935<br>Cl 0.0000000000 0.0000000000 1.7604519169<br>H -1.4303183434 0.0000000000 -0.6732889946<br>H 0.7151591717 1.2386920209 -0.6732889946<br>H 0.7151591717 -1.2386920209 -0.6732889946<br>N -0.0000000000 0.0000000000 -2.9248141834<br>H 0.9397426483 0.0000000000 -3.3029053979<br>H -0.4698713241 -0.8138410064 -3.3029053979<br>H -0.4698713241 0.8138410064 -3.3029053979
$ClSiH_3$···CNH	CCSD(T)-F12C/CC-PVDZ-F12 ENERGY=-843.94100284<br>Si -0.0000000000 0.0000000000 0.3050918930<br>Cl -0.0000000000 0.0000000000 2.3774923825<br>H 1.4092411139 0.0000000000 -0.1143269332<br>H -0.7046205570 -1.2204386047 -0.1143269332<br>H -0.7046205570 1.2204386047 -0.1143269332<br>C 0.0000000000 0.0000000000 -2.7368090099<br>N 0.0000000000 0.0000000000 -3.9052046698<br>H 0.0000000000 0.0000000000 -4.9024232253

$ClSiH_3 \cdots NP$	CCSD(T)-F12C/CC-PVDZ-F12 ENERGY=-1146.23868728<br>P 0.0000000000 0.0000000000 -4.7630478193<br>N -0.0000000000 0.0000000000 -3.2726419035<br>Si 0.0000000000 0.0000000000 -0.3718409002<br>H 0.7053615587 -1.2217220575 -0.7848946045<br>H -1.4107231175 0.0000000000 -0.7848946045<br>H 0.7053615587 1.2217220575 -0.7848946045<br>Cl -0.0000000000 0.0000000000 1.7023677330
$ClSiH_3 \cdots CS$	CCSD(T)-F12C/CC-PVDZ-F12 ENERGY=-1186.37982983<br>S 0.0000000000 0.0000000000 -4.9046623363<br>C 0.0000000000 0.0000000000 -3.3712060383<br>Si -0.0000000000 0.0000000000 -0.3161211949<br>H 0.7040615549 -1.2194703849 -0.7406351749<br>H -1.4081231099 0.0000000000 -0.7406351749<br>H 0.7040615549 1.2194703849 -0.7406351749<br>Cl 0.0000000000 0.0000000000 1.7540483906

NCSiH3···B, B= N2, CO, HCCH, PH3, HCN, H2CCH2, HNC, H2O, NH3, CS, NP

$NCSiH_3 \cdots N_2$	CCSD(T)-F12C/CC-PVDZ-F12 ENERGY=-492.98919289<br>N -0.0000040734 0.0000000000 -4.8672607066<br>N 0.0000101588 0.0000000000 -3.7685021357<br>Si -0.0000095619 0.0000000000 -0.4471151550<br>H 0.7028468240 -1.2173234583 -0.8822269873<br>H -1.4056988458 0.0000000000 -0.8822360817<br>H 0.7028468240 1.2173234583 -0.8822269873<br>C 0.0000002783 0.0000000000 1.4114636327<br>N 0.0000083961 0.0000000000 2.5724959302
$NCSiH_3 \cdots CO$	CCSD(T)-F12C/CC-PVDZ-F12 ENERGY=-496.77229269<br>O -0.0002012086 0.0000000000 -4.9266685353<br>C -0.0000908831 0.0000000000 -3.7974463197<br>Si 0.0004166289 0.0000000000 -0.4353715929<br>H 0.7032565052 -1.2187863722 -0.8655401156<br>H -1.4064170623 0.0000000000 -0.8658875224<br>H 0.7032565052 1.2187863722 -0.8655401156<br>C 0.0000129198 0.0000000000 1.4248790623<br>N -0.0002334050 0.0000000000 2.5859666484
$NCSiH_3 \cdots HCCH$	CCSD(T)-F12C/CC-PVDZ-F12 ENERGY=-460.79356413<br>Si 0.0028646186 0.8365575936 0.0000000000<br>C 0.0250671437 2.6978124501 0.0000000000<br>N 0.0386892623 3.8588672730 0.0000000000<br>H 0.6996971115 0.4023478693 1.2203679664<br>H 0.6996971115 0.4023478693 -1.2203679664<br>H -1.4108908973 0.4289478501 0.0000000000<br>C 0.5356134209 -2.6110359480 0.0000000000<br>C -0.6702334164 -2.6043289343 0.0000000000<br>H 1.5993131732 -2.6279466682 0.0000000000<br>H -1.7340125064 -2.6068689574 0.0000000000
$NCSiH_3 \cdots PH_3$	CCSD(T)-F12C/CC-PVDZ-F12 ENERGY=-726.29094112<br>Si 0.0000000000 0.0000000000 0.6610734618<br>C 0.0000000000 0.0000000000 2.5258537804

	H -1.4097791032 0.0000000000 0.2419142535<br>H 0.7048895516 1.2209045171 0.2419142535<br>H 0.7048895516 -1.2209045171 0.2419142535<br>P -0.0000000000 0.0000000000 -2.9123284742<br>H 1.1963820180 0.0000000000 -3.6621819020<br>H -0.5981910090 -1.0360972202 -3.6621819020<br>H -0.5981910090 1.0360972202 -3.6621819020<br>N 0.0000000000 0.0000000000 3.6870518006
$NCSiH_3$···NCH	CCSD(T)-F12C/CC-PVDZ-F12 ENERGY=-476.88906916<br>H -0.0000210618 0.0000000000 -5.7054211933<br>C 0.0000007687 0.0000000000 -4.6381114493<br>N 0.0000243488 0.0000000000 -3.4842890244<br>Si 0.0000276515 0.0000000000 -0.4793729553<br>H 0.7066076746 -1.2239365849 -0.8822071664<br>H -1.4132445278 0.0000000000 -0.8822318260<br>H 0.7066076746 1.2239365849 -0.8822071664<br>C 0.0000040078 0.0000000000 1.3894133202<br>N -0.0000065365 0.0000000000 2.5507591812
$NCSiH_3$··H_2CCH_2	CCSD(T)-F12C/CC-PVDZ-F12 ENERGY=-462.04577649<br>Si 0.0215476319 1.0186382432 0.0000000000<br>C 0.0076921267 2.8803906838 0.0000000000<br>N -0.0005360275 4.0414464263 0.0000000000<br>H -1.3842347927 0.5845344063 0.0000000000<br>H 0.7287698279 0.5975772294 1.2194494233<br>C -0.0255155009 -2.4106598751 0.6672576939<br>C -0.0255155009 -2.4106598751 -0.6672576939<br>H -0.9419045093 -2.2924253933 1.2316277281<br>H 0.8893824790 -2.5370908975 1.2317641703<br>H 0.8893824790 -2.5370908975 -1.2317641703<br>H -0.9419045093 -2.2924253933 -1.2316277281<br>H 0.7287698279 0.5975772294 -1.2194494233
$NCSiH_3$···OH_2	CCSD(T)-F12C/CC-PVDZ-F12 ENERGY=-459.94930739<br>O -0.1152437240 -0.0070892086 -2.9659081173<br>H -0.2879367447 -0.7799701928 -3.5062750555<br>H -0.3827096394 0.7384281370 -3.5061211975<br>Si -0.0614179034 -0.0038370909 -0.1343120795<br>H 0.6931332427 -1.1867759341 -0.5704598119<br>H -1.4968125450 -0.0940877504 -0.4372587971<br>H 0.5391571373 1.2642612110 -0.5703592634<br>C 0.0520322739 0.0032157744 1.7317031756<br>N 0.1213051175 0.0075197341 2.8909529085
$NCSiH_3$···NH_3	CCSD(T)-F12C/CC-PVDZ-F12 ENERGY=-440.08513523<br>N -0.0002212956 -0.0000027186 -2.8977710063<br>H 0.9364871420 0.0588735263 -3.2792225128<br>H -0.4176319853 -0.8407024456 -3.2790809276<br>H -0.5196454894 0.7818153502 -3.2791568851<br>Si 0.0004230601 0.0001336916 -0.2480922098<br>H 0.7924909275 -1.1933785839 -0.5750835238<br>H -1.4290510084 -0.0893523628 -0.5757285581<br>H 0.6373774131 1.2832423015 -0.5745914775<br>C -0.0000666435 -0.0000617065 1.6381174008

	N -0.0003385468 -0.0002560069 2.7999869465
$NCSiH_3\cdots CNH$	CCSD(T)-F12C/CC-PVDZ-F12 ENERGY=-476.86543138<br>Si -0.0000000000 0.0000000000 0.4617604755<br>C -0.0000000000 0.0000000000 2.3314163658<br>H 1.4141308186 0.0000000000 0.0618020364<br>H -0.7070654093 -1.2246732132 0.0618020364<br>H -0.7070654093 1.2246732132 0.0618020364<br>C 0.0000000000 0.0000000000 -2.6469870393<br>N 0.0000000000 0.0000000000 -3.8150733295<br>H 0.0000000000 0.0000000000 -4.8123992440<br>N -0.0000000000 0.0000000000 3.4927473701
$NCSiH_3\cdots NP$	CCSD(T)-F12C/CC-PVDZ-F12 ENERGY=-779.16338630<br>Si -0.0000000000 0.0000000000 1.1117696821<br>C -0.0000000000 0.0000000000 2.9835461302<br>H 1.4165126954 0.0000000000 0.7214342704<br>H -0.7082563477 -1.2267359790 0.7214342704<br>H -0.7082563477 1.2267359790 0.7214342704<br>N 0.0000000000 0.0000000000 -1.8039397684<br>P 0.0000000000 0.0000000000 -3.2941209476<br>N -0.0000000000 0.0000000000 4.1449511623
$NCSiH_3\cdots CS$	CCSD(T)-F12C/CC-PVDZ-F12 ENERGY=-819.30399456<br>Si -0.0000000000 0.0000000000 1.2123231518<br>C -0.0000000000 0.0000000000 3.0812777843<br>H 1.4134800400 0.0000000000 0.8088366624<br>H -0.7067400200 -1.2241096224 0.8088366624<br>H -0.7067400200 1.2241096224 0.8088366624<br>C 0.0000000000 0.0000000000 -1.9012155010<br>S 0.0000000000 0.0000000000 -3.4339644814<br>N -0.0000000000 0.0000000000 4.2425906768

Table S2. Optimised geometries (Å) and energies (au) of XGeH3···B complexes calculated at the CCSD(T)(F12c)/cc-pVDZ-F12 level. X= H, F, Cl and CN; B= N2, CO, HCCH, PH3, HCN, H2CCH2, HNC, H2O, NH3, CS, NP

HGeH3···B, B= N2, CO, HCCH, PH3, HCN, H2CCH2, HNC, H2O, NH3, CS, NP

$HGeH_3 \cdots N_2$	CCSD(T)-F12C/USERDEF ENERGY=-405.28756731			
	Ge	-0.0000000000	0.0000000000	1.1218317155
	H	-0.0000000000	0.0000000000	2.6563194112
	H	1.4462553078	0.0000000000	0.6128372357
	H	-0.7231276539	-1.2524938369	0.6128372357
	H	-0.7231276539	1.2524938369	0.6128372357
	N	0.0000000000	0.0000000000	-2.5191608980
	N	0.0000000000	0.0000000000	-3.6182079542
$HGeH_3 \cdots CO$	CCSD(T)-F12C/USERDEF ENERGY=-409.07018820			
	Ge	-0.0000000000	0.0000000000	1.1711052248
	H	-0.0000000000	0.0000000000	2.7063355918
	H	1.4469674682	0.0000000000	0.6640442200
	H	-0.7234837341	-1.2531105859	0.6640442200
	H	-0.7234837341	1.2531105859	0.6640442200
	C	0.0000000000	0.0000000000	-2.5584398290
	O	0.0000000000	0.0000000000	-3.6886934243
$HGeH_3 \cdots HCCH$	CCSD(T)-F12C/USERDEF ENERGY=-373.09109490			
	Ge	0.0123782487	1.0589519379	0.0000000000
	H	0.0079085890	2.5950443255	0.0000000000
	H	0.7356932871	0.5558711988	1.2544556018
	H	0.7356932871	0.5558711988	-1.2544556018
	H	-1.4350375224	0.5517260446	0.0000000000
	C	0.5349105119	-2.7228741875	0.0000000000
	C	-0.6703251721	-2.6969957726	0.0000000000
	H	1.5980229105	-2.7464183619	0.0000000000
	H	-1.7334391194	-2.6744759795	0.0000000000
$HGeH_3 \cdots PH_3$	CCSD(T)-F12C/USERDEF ENERGY=-638.58797828			
	Ge	0.0000000000	0.0000000000	1.2512962181
	H	0.0000000000	0.0000000000	2.7879388339
	H	-1.4479447002	0.0000000000	0.7473668510
	H	0.7239723501	1.2539568937	0.7473668510
	H	0.7239723501	-1.2539568937	0.7473668510
	P	-0.0000000000	0.0000000000	-2.7531741524
	H	1.1914114405	0.0000000000	-3.5139045776
	H	-0.5957057202	-1.0317925738	-3.5139045776
	H	-0.5957057202	1.0317925738	-3.5139045776
$HGeH_3 \cdots NCH$	CCSD(T)-F12C/USERDEF ENERGY=-389.18414094			
	Ge	-0.0000000000	0.0000000000	1.0507054391
	H	-0.0000000000	0.0000000000	2.5894836486
	H	1.4500427376	0.0000000000	0.5562205538
	H	-0.7250213688	-1.2557738473	0.5562205538
	H	-0.7250213688	1.2557738473	0.5562205538
	N	0.0000000000	0.0000000000	-2.3848903211
	C	0.0000000000	0.0000000000	-3.5396829073

	H 0.0000000000 0.0000000000 -4.6065124417
$HGeH_3 \cdot\cdot H_2CCH_2$	CCSD(T)-F12C/USERDEF ENERGY=-374.34319871<br>Ge 0.0157446690 1.2291976837 0.0000000000<br>H 0.0075247616 2.7653751401 0.0000000000<br>H -1.4296634575 0.7170184939 0.0000000000<br>H 0.7407686082 0.7266475049 1.2541500671<br>C -0.0189029558 -2.5258675733 0.6667710666<br>C -0.0189029558 -2.5258675733 -0.6667710666<br>H -0.9315227307 -2.3863697825 1.2316437445<br>H 0.8953241049 -2.6612484305 1.2298919080<br>H 0.8953241049 -2.6612484305 -1.2298919080<br>H -0.9315227307 -2.3863697825 -1.2316437445<br>H 0.7407686082 0.7266475049 -1.2541500671
$HGeH_3 \cdots OH_2$	CCSD(T)-F12C/USERDEF ENERGY=-372.24424180<br>Ge -0.0004864349 0.0008425300 -0.0201727962<br>H 0.0019443735 -0.0033677538 1.5187302018<br>H 1.4524453095 -0.0047119491 -0.5095815470<br>H -0.7221419871 -1.2602105102 -0.5095815470<br>H -0.7245672357 1.2549872658 -0.5149927686<br>O 0.0042395728 -0.0073431555 -3.2962526406<br>H 0.8860493004 -0.0184438050 -3.6722135237<br>H -0.4270518465 -0.7765631057 -3.6722135238
$HGeH_3 \cdots NH_3$	CCSD(T)-F12C/USERDEF ENERGY=-352.37755136<br>Ge -0.0000000000 0.0000000000 0.6202891703<br>H -0.0000000000 0.0000000000 2.1617718070<br>H 1.4535032609 0.0000000000 0.1367716140<br>H -0.7267516305 -1.2587707484 0.1367716140<br>H -0.7267516305 1.2587707484 0.1367716140<br>N 0.0000000000 0.0000000000 -2.7280414762<br>H 0.9370513507 0.0000000000 -3.1114422631<br>H -0.4685256754 -0.8115102744 -3.1114422631<br>H -0.4685256754 0.8115102744 -3.1114422631
$HGeH_3 \cdots CNH$	CCSD(T)-F12C/USERDEF ENERGY=-389.16036837<br>Ge -0.0000000000 0.0000000000 1.1150727387<br>H -0.0000000000 0.0000000000 2.6539738743<br>H 1.4499537454 0.0000000000 0.6201498481<br>H -0.7249768727 -1.2556967779 0.6201498481<br>H -0.7249768727 1.2556967779 0.6201498481<br>C 0.0000000000 0.0000000000 -2.4760939993<br>N 0.0000000000 0.0000000000 -3.6463415081<br>H 0.0000000000 0.0000000000 -4.6429563405
$HGeH_3 \cdots NP$	CCSD(T)-F12C/USERDEF ENERGY=-691.45773731<br>Ge -0.0000000000 0.0000000000 1.6310856884<br>H -0.0000000000 0.0000000000 3.1708504806<br>H 1.4510566983 0.0000000000 1.1393732338<br>H -0.7255283491 -1.2566519630 1.1393732338<br>H -0.7255283491 1.2566519630 1.1393732338<br>N 0.0000000000 0.0000000000 -1.7523267070<br>P 0.0000000000 0.0000000000 -3.2446006633
$HGeH_3 \cdots CS$	CCSD(T)-F12C/USERDEF ENERGY=-731.59949922<br>Ge -0.0000000000 0.0000000000 1.7182552207

	H -0.0000000000 0.0000000000 3.2563829759<br>H 1.4492042758 0.0000000000 1.2198708924<br>H -0.7246021379 -1.2550477181 1.2198708924<br>H -0.7246021379 1.2550477181 1.2198708924<br>C 0.0000000000 0.0000000000 -1.8703690721<br>S 0.0000000000 0.0000000000 -3.4071756770

FGeH3···B, B= N2, CO, HCCH, PH3, HCN, H2CCH2, HNC, H2O, NH3, CS, NP

$FGeH_3 \cdots N_2$	CCSD(T)-F12C/USERDEF ENERGY=-504.49693840<br>Ge -0.0000000000 0.0000000000 0.5851529951<br>F -0.0000000000 0.0000000000 2.3907345795<br>H 1.4792831495 0.0000000000 0.1995655043<br>H -0.7396415748 -1.2810967869 0.1995655043<br>H -0.7396415748 1.2810967869 0.1995655043<br>N 0.0000000000 0.0000000000 -2.6098296216<br>N 0.0000000000 0.0000000000 -3.7085640819
$FGeH_3 \cdots CO$	CCSD(T)-F12C/USERDEF ENERGY=-508.28046566<br>Ge -0.0000000000 0.0000000000 0.6027466028<br>F -0.0000000000 0.0000000000 2.4096768799<br>H 1.4807124081 0.0000000000 0.2239899525<br>H -0.7403562041 -1.2823345611 0.2239899525<br>H -0.7403562041 1.2823345611 0.2239899525<br>C 0.0000000000 0.0000000000 -2.5758294020<br>O 0.0000000000 0.0000000000 -3.7046643445
$FGeH_3 \cdots HCCH$	CCSD(T)-F12C/USERDEF ENERGY=-472.30169881<br>Ge 0.0114814963 0.7896198466 0.0000000000<br>F 0.0354193380 2.5980369172 0.0000000000<br>H 0.7455210950 0.4053142589 1.2837984052<br>H 0.7455210950 0.4053142589 -1.2837984052<br>H -1.4759893696 0.4359470848 0.0000000000<br>C 0.5340000754 -2.5369759591 0.0000000000<br>C -0.6720856459 -2.5330227485 0.0000000000<br>H 1.5978065039 -2.5502143547 0.0000000000<br>H -1.7358695691 -2.5373188972 0.0000000000
$FGeH_3 \cdots PH_3$	CCSD(T)-F12C/USERDEF ENERGY=-737.79948238<br>Ge 0.0000000000 0.0000000000 0.6593505654<br>F 0.0000000000 0.0000000000 2.4703237290<br>H -1.4839138601 0.0000000000 0.2937862564<br>H 0.7419569300 1.2851070998 0.2937862564<br>H 0.7419569300 -1.2851070998 0.2937862564<br>P -0.0000000000 0.0000000000 -2.7481580623<br>H 1.1988170148 0.0000000000 -3.4929432901<br>H -0.5994085074 -1.0382059893 -3.4929432901<br>H -0.5994085074 1.0382059893 -3.4929432901
$FGeH_3 \cdots NCH$	CCSD(T)-F12C/USERDEF ENERGY=-488.39750026<br>Ge -0.0000000000 0.0000000000 0.4984013196<br>F -0.0000000000 0.0000000000 2.3141089784<br>H 1.4863908877 0.0000000000 0.1498616160<br>H -0.7431954438 -1.2872522687 0.1498616160<br>H -0.7431954438 1.2872522687 0.1498616160

	N 0.0000000000 0.0000000000 -2.3868496505<br>C 0.0000000000 0.0000000000 -3.5401396679<br>H 0.0000000000 0.0000000000 -4.6073940385
$FGeH_3{\cdot\cdot}H_2CCH_2$	CCSD(T)-F12C/USERDEF ENERGY=-473.55413769<br>Ge 0.0147586913 0.9104324555 0.0000000000<br>F -0.0203030492 2.7191926929 0.0000000000<br>H -1.4608002911 0.5100968815 0.0000000000<br>H 0.7638079007 0.5508330646 1.2828957123<br>C -0.0150750055 -2.3800203026 0.6674544977<br>C -0.0150750055 -2.3800203026 -0.6674544977<br>H -0.9277690938 -2.2357429591 1.2313471553<br>H 0.8963228924 -2.5311626812 1.2310868148<br>H 0.8963228924 -2.5311626812 -1.2310868148<br>H -0.9277690938 -2.2357429591 -1.2313471553<br>H 0.7638079007 0.5508330646 -1.2828957123
$FGeH_3{\cdots}OH_2$	CCSD(T)-F12C/USERDEF ENERGY=-471.45783146<br>Ge 0.0067075172 -0.0116177605 -0.2852283427<br>F 0.0008718253 -0.0015100458 1.5298699326<br>H 1.4980300979 -0.0182299131 -0.6158727720<br>H -0.7332274815 -1.3064470767 -0.6158727720<br>H -0.7347454614 1.2726164702 -0.6396292235<br>O 0.0298558963 -0.0517119292 -3.0452524646<br>H 0.8609867565 0.0309838586 -3.5162343658<br>H -0.4573261871 -0.7301444739 -3.5162343658
$FGeH_3{\cdots}NH_3$	CCSD(T)-F12C/USERDEF ENERGY=-451.59496058<br>Ge 0.0000000000 0.0000000000 0.1076686351<br>F 0.0000000000 0.0000000000 1.9321314783<br>H -1.5005546101 0.0000000000 -0.1769190815<br>H 0.7502773050 1.2995184121 -0.1769190815<br>H 0.7502773050 -1.2995184121 -0.1769190815<br>N -0.0000000000 0.0000000000 -2.5154750298<br>H 0.9390638287 0.0000000000 -2.8952130538<br>H -0.4695319143 -0.8132531314 -2.8952130538<br>H -0.4695319143 0.8132531314 -2.8952130538
$FGeH_3{\cdots}CNH$	CCSD(T)-F12C/USERDEF ENERGY=-488.37435640<br>Ge -0.0000000000 0.0000000000 0.5303045773<br>F -0.0000000000 0.0000000000 2.3469003766<br>H 1.4879810010 0.0000000000 0.1875408133<br>H -0.7439905005 -1.2886293472 0.1875408133<br>H -0.7439905005 1.2886293472 0.1875408133<br>C 0.0000000000 0.0000000000 -2.4095229219<br>N 0.0000000000 0.0000000000 -3.5767268443<br>H 0.0000000000 0.0000000000 -4.5740206541
$FGeH_3{\cdots}NP$	CCSD(T)-F12C/USERDEF ENERGY=-790.67210205<br>Ge -0.0000000000 0.0000000000 0.9927905964<br>F -0.0000000000 0.0000000000 2.8113743896<br>H 1.4896707498 0.0000000000 0.6583142577<br>H -0.7448353749 -1.2900927126 0.6583142577<br>H -0.7448353749 1.2900927126 0.6583142577<br>N 0.0000000000 0.0000000000 -1.8083636453<br>P 0.0000000000 0.0000000000 -3.2976206239

$FGeH_3\cdots CS$	CCSD(T)-F12C/USERDEF ENERGY=-830.81297209<br>Ge -0.0000000000 0.0000000000 1.0449732929<br>F -0.0000000000 0.0000000000 2.8600090505<br>H 1.4877453532 0.0000000000 0.6989864067<br>H -0.7438726766 -1.2884252702 0.6989864067<br>H -0.7438726766 1.2884252702 0.6989864067<br>C 0.0000000000 0.0000000000 -1.8880866931<br>S 0.0000000000 0.0000000000 -3.4194016113

ClGeH3···B, B= N2, CO, HCCH, PH3, HCN, H2CCH2, HNC, H2O, NH3, CS, NP

$ClGeH_3\cdots N_2$	CCSD(T)-F12C/USERDEF ENERGY=-864.49132748<br>Ge -0.0000000000 0.0000000000 0.2365199148<br>Cl -0.0000000000 0.0000000000 2.4017194279<br>H 1.4637119173 0.0000000000 -0.2033894846<br>H -0.7318559586 -1.2676117042 -0.2033894846<br>H -0.7318559586 1.2676117042 -0.2033894846<br>N 0.0000000000 0.0000000000 -3.0810737364<br>N 0.0000000000 0.0000000000 -4.1798889673
$ClGeH_3\cdots CO$	CCSD(T)-F12C/USERDEF ENERGY=-868.27451844<br>Ge -0.0000000000 0.0000000000 0.2583128422<br>Cl -0.0000000000 0.0000000000 2.4262209850<br>H 1.4650090796 0.0000000000 -0.1763270058<br>H -0.7325045398 -1.2687350797 -0.1763270058<br>H -0.7325045398 1.2687350797 -0.1763270058<br>C 0.0000000000 0.0000000000 -3.0762703485<br>O 0.0000000000 0.0000000000 -4.2055000511
$ClGeH_3\cdots HCCH$	CCSD(T)-F12C/USERDEF ENERGY=-832.29580746<br>Ge 0.0087496662 0.8193387926 0.0000000000<br>Cl 0.0497298310 2.9890500670 0.0000000000<br>H 0.7312341018 0.3757283193 1.2704796767<br>H 0.7312341018 0.3757283193 -1.2704796767<br>H -1.4658738146 0.4176700416 0.0000000000<br>C 0.5356593241 -2.6246739936 0.0000000000<br>C -0.6702643352 -2.6172562053 0.0000000000<br>H 1.5993077624 -2.6406649088 0.0000000000<br>H -1.7339716221 -2.6182200250 0.0000000000
$ClGeH_3\cdots PH_3$	CCSD(T)-F12C/USERDEF ENERGY=-1097.79317870<br>Ge -0.0000000000 0.0000000000 0.3333625160<br>Cl -0.0000000000 0.0000000000 2.5069681583<br>H 1.4674825729 0.0000000000 -0.0913562129<br>H -0.7337412865 -1.2708771878 -0.0913562129<br>H -0.7337412865 1.2708771878 -0.0913562129<br>P 0.0000000000 0.0000000000 -3.2512265058<br>H 1.1962978497 0.0000000000 -4.0013535565<br>H -0.5981489248 -1.0360243283 -4.0013535565<br>H -0.5981489248 1.0360243283 -4.0013535565
$ClGeH_3\cdots NCH$	CCSD(T)-F12C/USERDEF ENERGY=-848.39096604<br>Ge -0.0000000000 0.0000000000 0.1556040307<br>Cl -0.0000000000 0.0000000000 2.3371130881<br>H 1.4710498676 0.0000000000 -0.2500420116

	H -0.7355249338 -1.2739665556 -0.2500420116<br>H -0.7355249338 1.2739665556 -0.2500420116<br>N 0.0000000000 0.0000000000 -2.8602450567<br>C 0.0000000000 0.0000000000 -4.0140401677<br>H 0.0000000000 0.0000000000 -5.0813083837
$ClGeH_3$··H_2CCH_2	CCSD(T)-F12C/USERDEF ENERGY=-833.54814756<br>Ge 0.0127812900 0.9608388263 0.0000000000<br>Cl 0.0005987661 3.1320709016 0.0000000000<br>H -1.4519666800 0.5250426365 0.0000000000<br>H 0.7474705024 0.5362139896 1.2709133838<br>C -0.0163152641 -2.4499726621 0.6672729033<br>C -0.0163152641 -2.4499726621 -0.6672729033<br>H -0.9301854385 -2.3156013768 1.2317641419<br>H 0.8971813683 -2.5897820701 1.2305312816<br>H 0.8971813683 -2.5897820701 -1.2305312816<br>H -0.9301854385 -2.3156013768 -1.2317641419<br>H 0.7474705024 0.5362139896 -1.2709133838
$ClGeH_3$···OH_2	CCSD(T)-F12C/USERDEF ENERGY=-831.45114794<br>Ge -0.0169031117 0.0292768386 0.1592727971<br>Cl 0.0575152386 -0.0996187305 2.3365635911<br>H 1.4410303608 0.0582308947 -0.2885950071<br>H -0.7709450160 -1.2188535566 -0.2885945377<br>H -0.7658108060 1.3264230869 -0.1348423014<br>O -0.0491217817 0.0850813895 -2.7168669850<br>H -0.8420565804 -0.0619735669 -3.2355174327<br>H 0.4746989854 0.6982561801 -3.2355168148
$ClGeH_3$···NH_3	CCSD(T)-F12C/USERDEF ENERGY=-811.58687527<br>Ge 0.0000000000 0.0000000000 -0.2221026954<br>Cl 0.0000000000 0.0000000000 1.9787323373<br>H -1.4838124803 0.0000000000 -0.5749868301<br>H 0.7419062401 1.2850193024 -0.5749868301<br>H 0.7419062401 -1.2850193024 -0.5749868301<br>N -0.0000000000 0.0000000000 -3.0026120581<br>H 0.9381215515 0.0000000000 -3.3845334394<br>H -0.4690607757 -0.8124370954 -3.3845334394<br>H -0.4690607757 0.8124370954 -3.3845334394
$ClGeH_3$···CNH	CCSD(T)-F12C/USERDEF ENERGY=-848.36751326<br>Ge -0.0000000000 0.0000000000 0.1888362980<br>Cl -0.0000000000 0.0000000000 2.3727166006<br>H 1.4719409787 0.0000000000 -0.2135696230<br>H -0.7359704894 -1.2747382804 -0.2135696230<br>H -0.7359704894 1.2747382804 -0.2135696230<br>C 0.0000000000 0.0000000000 -2.9097806237<br>N 0.0000000000 0.0000000000 -4.0778373425<br>H 0.0000000000 0.0000000000 -5.0750372443
$ClGeH_3$···NP	CCSD(T)-F12C/USERDEF ENERGY=-1150.66530047<br>Ge -0.0000000000 0.0000000000 0.6532288101<br>Cl -0.0000000000 0.0000000000 2.8398419888<br>H 1.4738079526 0.0000000000 0.2591887172<br>H -0.7369039763 -1.2763551272 0.2591887172<br>H -0.7369039763 1.2763551272 0.2591887172

	N 0.0000000000 0.0000000000 -2.2838561374<br>P 0.0000000000 0.0000000000 -3.7739444492
$ClGeH_3\cdots CS$	CCSD(T)-F12C/USERDEF ENERGY=-1190.80618133<br>Ge -0.0000000000 0.0000000000 0.7070507713<br>Cl -0.0000000000 0.0000000000 2.8886470560<br>H 1.4713338917 0.0000000000 0.3003079791<br>H -0.7356669459 -1.2742125277 0.3003079791<br>H -0.7356669459 1.2742125277 0.3003079791<br>C 0.0000000000 0.0000000000 -2.3939223136<br>S 0.0000000000 0.0000000000 -3.9267216448

NCGeH3···B, B= N2, CO, HCCH, PH3, HCN, H2CCH2, HNC, H2O, NH3, CS, NP

$NCGeH_3\cdots N_2$	CCSD(T)-F12C/USERDEF ENERGY=-497.41722066<br>Ge -0.0000000000 0.0000000000 0.5294566491<br>C -0.0000000000 0.0000000000 2.4705132344<br>H 1.4620154362 0.0000000000 0.0937689446<br>H -0.7310077181 -1.2661425083 0.0937689446<br>H -0.7310077181 1.2661425083 0.0937689446<br>N 0.0000000000 0.0000000000 -2.8666612457<br>N -0.0000000000 0.0000000000 -3.9655300094<br>N -0.0000000000 0.0000000000 3.6314344959
$NCGeH_3\cdots CO$	CCSD(T)-F12C/USERDEF ENERGY=-501.20029929<br>Ge 0.0000000000 0.0000000000 0.5739231264<br>C 0.0000000000 0.0000000000 2.5167063646<br>H 1.4629987623 0.0000000000 0.1423370650<br>H -0.7314993812 -1.2669940939 0.1423370650<br>H -0.7314993812 1.2669940939 0.1423370650<br>C 0.0000000000 0.0000000000 -2.8679911508<br>O -0.0000000000 0.0000000000 -3.9971974190<br>N 0.0000000000 0.0000000000 3.6776854364
$NCGeH_3\cdots HCCH$	CCSD(T)-F12C/USERDEF ENERGY=-465.22163346<br>Ge 0.0029343892 0.8525634996 0.0000000000<br>C 0.0267616131 2.7967034317 0.0000000000<br>N 0.0412266654 3.9576503011 0.0000000000<br>H 0.7271719430 0.4150761372 1.2684038411<br>H 0.7271719430 0.4150761372 -1.2684038411<br>H -1.4670351775 0.4438916024 0.0000000000<br>C 0.5348336766 -2.6740198388 0.0000000000<br>C -0.6709968882 -2.6673057306 0.0000000000<br>H 1.5984958301 -2.6918009820 0.0000000000<br>H -1.7347589732 -2.6711341604 0.0000000000
$NCGeH_3\cdots PH_3$	CCSD(T)-F12C/USERDEF ENERGY=-730.71897866<br>Ge -0.0000000000 0.0000000000 0.6898332946<br>C -0.0000000000 0.0000000000 2.6374239680<br>H -1.4652370477 0.0000000000 0.2669267968<br>H 0.7326185239 1.2689325059 0.2669267967<br>H 0.7326185239 -1.2689325059 0.2669267967<br>P -0.0000000000 0.0000000000 -2.9933130336<br>H 1.1958947219 0.0000000000 -3.7441444131<br>H -0.5979473610 -1.0356752094 -3.7441444131

	H -0.5979473610 1.0356752094 -3.7441444131<br>N 0.0000000000 0.0000000000 3.7985562431
$NCGeH_3\cdots NCH$	CCSD(T)-F12C/USERDEF ENERGY=-481.31714381<br>Ge 0.0000226466 0.0000000000 0.4129393141<br>C -0.0000204904 0.0000000000 2.3656734199<br>H 1.4682827263 0.0000000000 0.0060926346<br>H -0.7340742702 -1.2715378296 0.0061050279<br>H -0.7340742702 1.2715378296 0.0061050279<br>N -0.0000051461 0.0000000000 -2.6778054856<br>C -0.0000214726 0.0000000000 -3.8317517053<br>H -0.0000387340 0.0000000000 -4.8990610204<br>N -0.0000762812 0.0000000000 3.5269712617
$NCGeH_3\cdots H_2CCH_2$	CCSD(T)-F12C/USERDEF ENERGY=-466.47382391<br>Ge 0.0121401910 1.0411337078 0.0000000000<br>C 0.0169137140 2.9875776403 0.0000000000<br>N 0.0219497459 4.1486824410 0.0000000000<br>H -1.4530144908 0.6167908210 0.0000000000<br>H 0.7427995354 0.6116238211 1.2676229198<br>C -0.0230497134 -2.4605951586 0.6671866218<br>C -0.0230497134 -2.4605951586 -0.6671866218<br>H -0.9400423089 -2.3560285786 1.2330135431<br>H 0.8942646729 -2.5721864457 1.2307933993<br>H 0.8942646729 -2.5721864457 -1.2307933993<br>H -0.9400423089 -2.3560285786 -1.2330135431<br>H 0.7427995354 0.6116238211 -1.2676229198
$NCGeH_3\cdots OH_2$	CCSD(T)-F12C/USERDEF ENERGY=-464.37721318<br>Ge -0.0261096947 0.0452258692 0.2442638899<br>C 0.0228077919 -0.0394997985 2.1957955294<br>N 0.0503561462 -0.0872283094 3.3558059393<br>H 1.4339591870 0.0649807820 -0.1906908140<br>H -0.7732551370 -1.2093544986 -0.1906825613<br>H -0.7709897031 1.3353974091 -0.0765836202<br>O -0.0570066690 0.0987374249 -2.6901551764<br>H -0.8340322914 -0.0753784560 -3.2242506111<br>H 0.4822928102 0.6846086910 -3.2242469676
$NCGeH_3\cdots NH_3$	CCSD(T)-F12C/USERDEF ENERGY=-444.51209933<br>Ge 0.0000011332 0.0000000000 -0.1420701580<br>C 0.0000043213 0.0000000000 1.8240779113<br>H -1.4777589022 0.0000000000 -0.5118506111<br>H 0.7388636776 1.2797567619 -0.5118383499<br>H 0.7388636776 -1.2797567619 -0.5118383499<br>N -0.0000051222 0.0000000000 -3.0498814969<br>H 0.9368896141 0.0000000000 -3.4353617626<br>H -0.4684325731 -0.8113475556 -3.4353387183<br>H -0.4684325731 0.8113475556 -3.4353387183<br>N 0.0000120386 0.0000000000 2.9856889128
$NCGeH_3\cdots CNH$	CCSD(T)-F12C/USERDEF ENERGY=-481.29344119<br>Ge 0.0000000000 0.0000000000 0.4795120002<br>C 0.0000000000 0.0000000000 2.4333675077<br>H 1.4685858388 0.0000000000 0.0735905604<br>H -0.7342929194 -1.2718326441 0.0735905604

	H -0.7342929194 1.2718326441 0.0735905604<br>C 0.0000000000 0.0000000000 -2.7325580783<br>N 0.0000000000 0.0000000000 -3.9008304699<br>H -0.0000000000 0.0000000000 -4.8980909694<br>N 0.0000000000 0.0000000000 3.5946990365
$NCGeH_3$···NP	CCSD(T)-F12C/USERDEF ENERGY=-783.59134715<br>Ge -0.0000000000 0.0000000000 1.1208992180<br>C 0.0000000000 0.0000000000 3.0771757060<br>H 1.4703783484 0.0000000000 0.7227537555<br>H -0.7351891742 -1.2733850029 0.7227537555<br>H -0.7351891742 1.2733850029 0.7227537555<br>N 0.0000000000 0.0000000000 -1.9039998429<br>P -0.0000000000 0.0000000000 -3.3944165963<br>N -0.0000000000 0.0000000000 4.2385893184
$NCGeH_3$···CS	CCSD(T)-F12C/USERDEF ENERGY=-823.73194961<br>Ge 0.0000000000 0.0000000000 1.2217686102<br>C 0.0000000000 0.0000000000 3.1740004880<br>H 1.4679387915 0.0000000000 0.8119624914<br>H -0.7339693958 -1.2712722847 0.8119624914<br>H -0.7339693958 1.2712722847 0.8119624914<br>C 0.0000000000 0.0000000000 -2.0031630720<br>S 0.0000000000 0.0000000000 -3.5362562008<br>N 0.0000000000 0.0000000000 4.3352843181

Table S3. Optimised geometries (Å) and energies (au) of XSnH3···B complexes calculated at the CCSD(T)(F12c)/cc-pVDZ-F12 level. X= H, F, Cl and CN; B= N2, CO, HCCH, PH3, HCN, H2CCH2, HNC, H2O, NH3, CS, NP

HSnH3···B, B= N2, CO, HCCH, PH3, HCN, H2CCH2, HNC, H2O, NH3, CS, NP

$HSnH_3 \cdots N_2$	CCSD(T)-F12C/USERDEF ENERGY=-325.42479499<br>Sn 0.0000000000 0.0000000000 0.7860685241<br>H 0.0000000000 0.0000000000 2.4876857844<br>H 1.6049173343 0.0000000000 0.2218993805<br>H -0.8024586671 -1.3898991824 0.2218993805<br>H -0.8024586671 1.3898991824 0.2218993805<br>N -0.0000000000 0.0000000000 -2.9000054850<br>N 0.0000000000 0.0000000000 -3.9989372547
$HSnH_3 \cdots CO$	CCSD(T)-F12C/USERDEF ENERGY=-329.20749557<br>Sn -0.0000000000 0.0000000000 0.8167386336<br>H 0.0000000000 0.0000000000 2.5193400314<br>H 1.6059868344 0.0000000000 0.2562876718<br>H -0.8029934172 -1.3908253968 0.2562876718<br>H -0.8029934172 1.3908253968 0.2562876718<br>C 0.0000000000 0.0000000000 -2.9352435869<br>O -0.0000000000 0.0000000000 -4.0652024947
$HSnH_3 \cdots HCCH$	CCSD(T)-F12C/USERDEF ENERGY=-293.22857046<br>Sn 0.0041093565 1.0760033820 0.0000000000<br>H -0.0240569844 2.7793290380 0.0000000000<br>H 0.8192858446 0.5312596770 1.3902989950<br>H 0.8192858446 0.5312596770 -1.3902989950<br>H -1.5953494643 0.4997123170 0.0000000000<br>C 0.5427557846 -2.7637473440 0.0000000000<br>C -0.6615170051 -2.7072654794 0.0000000000<br>H 1.6050980206 -2.8151792660 0.0000000000<br>H -1.7238063834 -2.6546715880 0.0000000000
$HSnH_3 \cdots PH_3$	CCSD(T)-F12C/USERDEF ENERGY=-558.72547634<br>Sn -0.0000000000 0.0000000000 1.2586104255<br>H -0.0000000000 0.0000000000 2.9629199511<br>H -1.6080714176 0.0000000000 0.7042847985<br>H 0.8040357088 1.3926306988 0.7042847985<br>H 0.8040357088 -1.3926306988 0.7042847985<br>P -0.0000000000 0.0000000000 -2.7677682895<br>H 1.1922726730 0.0000000000 -3.5267229209<br>H -0.5961363365 -1.0325384230 -3.5267229209<br>H -0.5961363365 1.0325384230 -3.5267229209
$HSnH_3 \cdots NCH$	CCSD(T)-F12C/USERDEF ENERGY=-309.32193563<br>Sn -0.0000000000 0.0000000000 0.7217650494<br>H -0.0000000000 0.0000000000 2.4301296398<br>H 1.6126961384 0.0000000000 0.1843043940<br>H -0.8063480692 -1.3966358245 0.1843043940<br>H -0.8063480692 1.3966358245 0.1843043940<br>N -0.0000000000 0.0000000000 -2.6895192606<br>C -0.0000000000 0.0000000000 -3.8441095732

	H -0.0000000000 0.0000000000 -4.9110080726
$HSnH_3$··H_2CCH_2	CCSD(T)-F12C/USERDEF ENERGY=-294.48072528<br>Sn 0.0096790967 1.2386984104 0.0000000000<br>H -0.0372105064 2.9419308506 0.0000000000<br>H -1.5823165017 0.6412839580 0.0000000000<br>H 0.8300922500 0.7011880038 1.3907188659<br>C -0.0128019759 -2.5438753778 0.6668702504<br>C -0.0128019759 -2.5438753778 -0.6668702504<br>H -0.9143659498 -2.3433706333 1.2313976787<br>H 0.8898922503 -2.7413534317 1.2301297844<br>H 0.8898922503 -2.7413534317 -1.2301297844<br>H -0.9143659498 -2.3433706333 -1.2313976787<br>H 0.8300922500 0.7011880038 -1.3907188659
$HSnH_3$···OH_2	CCSD(T)-F12C/USERDEF ENERGY=-292.38238552<br>Sn 0.0086013160 0.0000000000 0.4042033837<br>H -0.0047404377 0.0000000000 2.1130420650<br>H 1.6208613622 0.0000000000 -0.1216839409<br>H -0.8119211672 -1.3936519302 -0.1277520537<br>H -0.8119211672 1.3936519302 -0.1277520537<br>O -0.0115179488 0.0000000000 -2.8072466836<br>H -0.4635980572 -0.7589455799 -3.1801703703<br>H -0.4635980572 0.7589455799 -3.1801703703
$HSnH_3$···NH_3	CCSD(T)-F12C/USERDEF ENERGY=-272.51624764<br>Sn -0.0000000000 0.0000000000 0.3988294787<br>H -0.0000000000 0.0000000000 2.1133274903<br>H 1.6232112787 0.0000000000 -0.1076110136<br>H -0.8116056394 -1.4057422031 -0.1076110136<br>H -0.8116056394 1.4057422031 -0.1076110136<br>N 0.0000000000 0.0000000000 -2.8177126739<br>H 0.9378341570 0.0000000000 -3.1995706391<br>H -0.4689170785 -0.8121882045 -3.1995706391<br>H -0.4689170785 0.8121882045 -3.1995706391
$HSnH_3$···CNH	CCSD(T)-F12C/USERDEF ENERGY=-309.29816362<br>Sn 0.0000000000 0.0000000000 0.7712346671<br>H -0.0000000000 0.0000000000 2.4800025579<br>H 1.6129520691 0.0000000000 0.2350430638<br>H -0.8064760346 -1.3968574670 0.2350430638<br>H -0.8064760346 1.3968574670 0.2350430638<br>C -0.0000000000 0.0000000000 -2.7866213650<br>N 0.0000000000 0.0000000000 -3.9564426305<br>H 0.0000000000 0.0000000000 -4.9531314560
$HSnH_3$···NP	CCSD(T)-F12C/USERDEF ENERGY=-611.59575821<br>Sn -0.0000000000 0.0000000000 1.1693357708<br>H -0.0000000000 0.0000000000 2.8795589739<br>H 1.6152621697 0.0000000000 0.6395365226<br>H -0.8076310848 -1.3988580727 0.6395365226<br>H -0.8076310848 1.3988580727 0.6395365226<br>N 0.0000000000 0.0000000000 -2.1658464015<br>P 0.0000000000 0.0000000000 -3.6575594260
$HSnH_3$···CS	CCSD(T)-F12C/USERDEF ENERGY=-651.73724272<br>Sn -0.0000000000 0.0000000000 1.2354942543

	H -0.0000000000 0.0000000000 2.9431964424<br>H 1.6115831974 0.0000000000 0.6931274056<br>H -0.8057915987 -1.3956719892 0.6931274056<br>H -0.8057915987 1.3956719892 0.6931274056<br>C 0.0000000000 0.0000000000 -2.3248839245<br>S 0.0000000000 0.0000000000 -3.8608574596

FSnH3···B, B= N2, CO, HCCH, PH3, HCN, H2CCH2, HNC, H2O, NH3, CS, NP

$FSnH_3 \cdots N_2$	CCSD(T)-F12C/USERDEF ENERGY=-424.64143676<br>Sn -0.0000000000 0.0000000000 0.4275543837<br>F 0.0000000000 0.0000000000 2.3479365975<br>H 1.6424261793 0.0000000000 0.0015242892<br>H -0.8212130897 -1.4223827951 0.0015242892<br>H -0.8212130897 1.4223827951 0.0015242892<br>N -0.0000000000 0.0000000000 -2.8493464912<br>N 0.0000000000 0.0000000000 -3.9480343793
$FSnH_3 \cdots CO$	CCSD(T)-F12C/USERDEF ENERGY=-428.42503586<br>Sn -0.0000000000 0.0000000000 0.4432368095<br>F 0.0000000000 0.0000000000 2.3662008236<br>H 1.6446975089 0.0000000000 0.0266224574<br>H -0.8223487544 -1.4243498241 0.0266224574<br>H -0.8223487544 1.4243498241 0.0266224574<br>C -0.0000000000 0.0000000000 -2.8216782516<br>O -0.0000000000 0.0000000000 -3.9501877369
$FSnH_3 \cdots HCCH$	CCSD(T)-F12C/USERDEF ENERGY=-392.44660892<br>Sn 0.0083952025 0.8145706172 0.0000000000<br>F 0.0132445235 2.7395309060 0.0000000000<br>H 0.8353061824 0.4027609120 1.4232208111<br>H 0.8353061824 0.4027609120 -1.4232208111<br>H -1.6412184322 0.4225492348 0.0000000000<br>C 0.5368356138 -2.5760173825 0.0000000000<br>C -0.6693762941 -2.5652831599 0.0000000000<br>H 1.6006318640 -2.5994987999 0.0000000000<br>H -1.7333198303 -2.5646728279 0.0000000000
$FSnH_3 \cdots PH_3$	CCSD(T)-F12C/USERDEF ENERGY=-657.94447785<br>Sn -0.0018040266 0.0000000000 0.4874234877<br>F -0.0033835132 0.0000000000 2.4161380290<br>H -1.6516196449 0.0000000000 0.0983306075<br>H 0.8291247616 1.4263513738 0.0924891916<br>H 0.8291247616 -1.4263513738 0.0924891916<br>P -0.0017017349 0.0000000000 -2.9854540368<br>H 1.2011074987 0.0000000000 -3.7227002574<br>H -0.6004240512 -1.0400566823 -3.7271615282<br>H -0.6004240512 1.0400566823 -3.7271615282
$FSnH_3 \cdots NCH$	CCSD(T)-F12C/USERDEF ENERGY=-408.54312733<br>Sn 0.0000000000 0.0000000000 0.3578194914<br>F 0.0000000000 0.0000000000 2.2917357411<br>H 1.6542446438 0.0000000000 -0.0122637454<br>H -0.8271223219 -1.4326178856 -0.0122637454<br>H -0.8271223219 1.4326178856 -0.0122637454

	N 0.0000000000 0.0000000000 -2.5732284738<br>C 0.0000000000 0.0000000000 -3.7262002759<br>H -0.0000000000 0.0000000000 -4.7936587102
$FSnH_3 \cdot\cdot H_2CCH_2$	CCSD(T)-F12C/USERDEF ENERGY=-393.69909827<br>Sn 0.0171565893 0.9418391141 0.0000000000<br>F -0.0061853347 2.8675600269 0.0000000000<br>H -1.6272580748 0.5287635405 0.0000000000<br>H 0.8494840650 0.5420497861 1.4242041680<br>C -0.0200644196 -2.4098612667 0.6676483384<br>C -0.0200644196 -2.4098612667 -0.6676483384<br>H -0.9362253946 -2.2912330196 1.2322826858<br>H 0.8948101308 -2.5377546137 1.2314181341<br>H 0.8948101308 -2.5377546137 -1.2314181341<br>H -0.9362253946 -2.2912330196 -1.2322826858<br>H 0.8494840650 0.5420497861 -1.4242041680
$FSnH_3 \cdots OH_2$	CCSD(T)-F12C/USERDEF ENERGY=-391.60389583<br>Sn -0.0313666379 0.0000000000 0.1664128047<br>F 0.0886067017 0.0000000000 2.0970796312<br>H -1.7089789108 0.0000000000 -0.0799885750<br>H 0.7699293906 1.4365228477 -0.2460871545<br>H 0.7699293906 -1.4365228477 -0.2460871545<br>O -0.0900674548 0.0000000000 -2.6086429845<br>H -0.3687876575 -0.7627215634 -3.1189423647<br>H -0.3687876575 0.7627215634 -3.1189423647
$FSnH_3 \cdots NH_3$	CCSD(T)-F12C/USERDEF ENERGY=-371.74189110<br>Sn -0.0000000000 0.0000000000 0.0725113657<br>F -0.0000000000 0.0000000000 2.0200483313<br>H -1.6721662099 0.0000000000 -0.2217884234<br>H 0.8360831049 1.4481384171 -0.2217884234<br>H 0.8360831049 -1.4481384171 -0.2217884234<br>N -0.0000000000 0.0000000000 -2.6074375270<br>H 0.9394440963 0.0000000000 -2.9867496465<br>H -0.4697220481 -0.8135824528 -2.9867496465<br>H -0.4697220481 0.8135824528 -2.9867496465
$FSnH_3 \cdots CNH$	CCSD(T)-F12C/USERDEF ENERGY=-408.51996765<br>Sn -0.0000000000 0.0000000000 0.3830433557<br>F 0.0000000000 0.0000000000 2.3193558734<br>H 1.6566243465 0.0000000000 0.0218027172<br>H -0.8283121733 -1.4346787686 0.0218027172<br>H -0.8283121733 1.4346787686 0.0218027172<br>C 0.0000000000 0.0000000000 -2.6124612883<br>N 0.0000000000 0.0000000000 -3.7787843830<br>H -0.0000000000 0.0000000000 -4.7762973440
$FSnH_3 \cdots NP$	CCSD(T)-F12C/USERDEF ENERGY=-710.81822614<br>Sn -0.0000000000 0.0000000000 0.7418134163<br>F -0.0000000000 0.0000000000 2.6803397871<br>H 1.6593629528 0.0000000000 0.3934396407<br>H -0.8296814764 -1.4370504712 0.3934396407<br>H -0.8296814764 1.4370504712 0.3934396407<br>N 0.0000000000 0.0000000000 -2.0911030243<br>P 0.0000000000 0.0000000000 -3.5794248988

$FSnH_3\cdots CS$	CCSD(T)-F12C/USERDEF ENERGY=-750.95846663<br>Sn -0.0000000000 0.0000000000 0.7847868785<br>F -0.0000000000 0.0000000000 2.7199322728<br>H 1.6564275376 0.0000000000 0.4196045879<br>H -0.8282137688 -1.4345083271 0.4196045879<br>H -0.8282137688 1.4345083271 0.4196045879<br>C 0.0000000000 0.0000000000 -2.2021386678<br>S 0.0000000000 0.0000000000 -3.7317418985

ClSnH3···B, B= N2, CO, HCCH, PH3, HCN, H2CCH2, HNC, H2O, NH3, CS, NP

$ClSnH_3\cdots N_2$	CCSD(T)-F12C/USERDEF ENERGY=-784.63627143<br>Sn -0.0000000000 0.0000000000 0.1644140072<br>Cl 0.0000000000 0.0000000000 2.4984266030<br>H 1.6314843607 0.0000000000 -0.2996804472<br>H -0.8157421803 -1.4129069022 -0.2996804472<br>H -0.8157421803 1.4129069022 -0.2996804472<br>N -0.0000000000 0.0000000000 -3.1817100825<br>N 0.0000000000 0.0000000000 -4.2804903813
$ClSnH_3\cdots CO$	CCSD(T)-F12C/USERDEF ENERGY=-788.41968653<br>Sn -0.0000000000 0.0000000000 0.1646841068<br>Cl 0.0000000000 0.0000000000 2.5022622260<br>H 1.6337310791 0.0000000000 -0.2897358224<br>H -0.8168655395 -1.4148526174 -0.2897358224<br>H -0.8168655395 1.4148526174 -0.2897358224<br>C -0.0000000000 0.0000000000 -3.1836543721<br>O 0.0000000000 0.0000000000 -4.3124856887
$ClSnH_3\cdots HCCH$	CCSD(T)-F12C/USERDEF ENERGY=-752.44124193<br>Sn 0.0104896417 0.8439516538 0.0000000000<br>Cl 0.0244470213 3.1844474622 0.0000000000<br>H 0.8281393596 0.3922643534 1.4153983188<br>H 0.8281393596 0.3922643534 -1.4153983188<br>H -1.6301383400 0.4182546209 0.0000000000<br>C 0.5342259076 -2.6154852093 0.0000000000<br>C -0.6717882817 -2.6026183744 0.0000000000<br>H 1.5978724855 -2.6381895907 0.0000000000<br>H -1.7355821504 -2.5981888555 0.0000000000
$ClSnH_3\cdots PH_3$	CCSD(T)-F12C/USERDEF ENERGY=-1017.93893446<br>Sn -0.0016801887 0.0000000000 0.4957038898<br>Cl -0.0032770518 0.0000000000 2.8412967179<br>H -1.6414973412 0.0000000000 0.0706356582<br>H 0.8244509343 1.4175680367 0.0653791603<br>H 0.8244509343 -1.4175680367 0.0653791603<br>P -0.0028917807 0.0000000000 -3.0485909752<br>H 1.1997882792 0.0000000000 -3.7868399548<br>H -0.5996718927 -1.0388703931 -3.7942852498<br>H -0.5996718927 1.0388703931 -3.7942852498
$ClSnH_3\cdots NCH$	CCSD(T)-F12C/USERDEF ENERGY=-768.53736683<br>Sn -0.0000000000 0.0000000000 0.0790615252<br>Cl -0.0000000000 0.0000000000 2.4335117273<br>H 1.6450198010 0.0000000000 -0.3261106788

	H -0.8225099005 -1.4246289374 -0.3261106788<br>H -0.8225099005 1.4246289374 -0.3261106788<br>N 0.0000000000 0.0000000000 -2.9077330801<br>C 0.0000000000 0.0000000000 -4.0609470064<br>H 0.0000000000 0.0000000000 -5.1285039469
$ClSnH_3$··H_2CCH_2	CCSD(T)-F12C/USERDEF ENERGY=-753.69369334<br>Sn 0.0172486266 0.9837757317 0.0000000000<br>Cl -0.0000010661 3.3252025837 0.0000000000<br>H -1.6172923023 0.5358202879 0.0000000000<br>H 0.8415983347 0.5442206552 1.4160062381<br>C -0.0257818501 -2.4338912189 0.6675140485<br>C -0.0257818501 -2.4338912189 -0.6675140485<br>H -0.9417867961 -2.3109933130 1.2313977065<br>H 0.8886698490 -2.5645617267 1.2313572859<br>H 0.8886698490 -2.5645617267 -1.2313572859<br>H -0.9417867961 -2.3109933130 -1.2313977065<br>H 0.8415983347 0.5442206552 -1.4160062381
$ClSnH_3$···OH_2	CCSD(T)-F12C/USERDEF ENERGY=-751.59805611<br>Sn -0.0523290499 0.0000000000 0.1780297106<br>Cl 0.1343976398 0.0000000000 2.5263955345<br>H -1.7259988697 0.0000000000 -0.0772486548<br>H 0.7347144222 1.4293443255 -0.2789195982<br>H 0.7347144222 -1.4293443255 -0.2789195982<br>O -0.1231953935 0.0000000000 -2.6341160161<br>H -0.3208359835 -0.7630585854 -3.1800927643<br>H -0.3208359835 0.7630585854 -3.1800927643
$ClSnH_3$···NH_3	CCSD(T)-F12C/USERDEF ENERGY=-731.73559008<br>Sn -0.0000000000 0.0000000000 0.0666692025<br>Cl -0.0000000000 0.0000000000 2.4457688047<br>H -1.6647204926 0.0000000000 -0.2568536013<br>H 0.8323602463 1.4416902368 -0.2568536013<br>H 0.8323602463 -1.4416902368 -0.2568536013<br>N 0.0000000000 0.0000000000 -2.6357855763<br>H 0.9393687960 0.0000000000 -3.0155278889<br>H -0.4696843980 -0.8135172409 -3.0155278889<br>H -0.4696843980 0.8135172409 -3.0155278889
$ClSnH_3$···CNH	Sn -0.0000000000 0.0000000000 0.1008949686<br>Cl -0.0000000000 0.0000000000 2.4592554937<br>H 1.6468812054 0.0000000000 -0.2962976360<br>H -0.8234406027 -1.4262409609 -0.2962976360<br>H -0.8234406027 1.4262409609 -0.2962976360<br>C 0.0000000000 0.0000000000 -2.9506811294<br>N 0.0000000000 0.0000000000 -4.1174008742<br>H 0.0000000000 0.0000000000 -5.1148166082
$ClSnH_3$···NP	CCSD(T)-F12C/USERDEF ENERGY=-1070.81235950<br>Sn 0.0000000000 0.0000000000 -0.0110984919<br>Cl -0.0000000000 0.0000000000 2.3513332122<br>H 1.6502263080 0.0000000000 -0.3928340809<br>H -0.8251131540 -1.4291379047 -0.3928340809<br>H -0.8251131540 1.4291379047 -0.3928340809<br>N -0.0000000000 0.0000000000 -2.8856850484

	P 0.0000000000 0.0000000000 -4.3744486245
$ClSnH_3\cdots CS$	CCSD(T)-F12C/USERDEF ENERGY=-1110.95264940<br>Sn -0.0000000000 0.0000000000 0.5030254498<br>Cl -0.0000000000 0.0000000000 2.8594449935<br>H 1.6462538820 0.0000000000 0.1008246708<br>H -0.8231269410 -1.4256976829 0.1008246708<br>H -0.8231269410 1.4256976829 0.1008246708<br>C 0.0000000000 0.0000000000 -2.5485432072<br>S 0.0000000000 0.0000000000 -4.0790491949

NCSnH3···B, B= N2, CO, HCCH, PH3, HCN, H2CCH2, HNC, H2O, NH3, CS, NP

$NCSnH_3\cdots N_2$	CCSD(T)-F12C/USERDEF ENERGY=-417.82950002<br>Sn -0.0000000000 0.0000000000 0.5006702787<br>C 0.0000000002 0.0000000000 2.6144040467<br>H 1.6219078431 0.0000000000 0.0395464363<br>H -0.8109539216 -1.4046133948 0.0395464362<br>H -0.8109539216 1.4046133948 0.0395464362<br>N 0.0000000000 0.0000000000 -2.9153634477<br>N -0.0000000000 0.0000000000 -4.0141529573<br>N -0.0000000000 0.0000000000 3.7763227290
$NCSnH_3\cdots CO$	CCSD(T)-F12C/USERDEF ENERGY=-421.61276856<br>Sn 0.0000000000 0.0000000000 0.5383454760<br>C -0.0000000000 0.0000000000 2.6552097640<br>H 1.6238964014 0.0000000000 0.0851950369<br>H -0.8119482008 -1.4063355367 0.0851950370<br>H -0.8119482008 1.4063355367 0.0851950370<br>C -0.0000000000 0.0000000000 -2.9036771923<br>O 0.0000000000 0.0000000000 -4.0325366286<br>N -0.0000000000 0.0000000000 3.8172110226
$NCSnH_3\cdots HCCH$	CCSD(T)-F12C/USERDEF ENERGY=-385.63435549<br>Sn 0.0036504164 0.8156356217 0.0000000000<br>C 0.0235633776 2.9347694448 0.0000000000<br>N 0.0382692554 4.0967575309 0.0000000000<br>H 0.8127631286 0.3650956989 1.4080435825<br>H 0.8127631286 0.3650956989 -1.4080435825<br>H -1.6274260851 0.3867340085 0.0000000000<br>C 0.5336145751 -2.6972607781 0.0000000000<br>C -0.6723865055 -2.6845604444 0.0000000000<br>H 1.5972984126 -2.7222508290 0.0000000000<br>H -1.7363046822 -2.6833155548 0.0000000000
$NCSnH_3\cdots PH_3$	CCSD(T)-F12C/USERDEF ENERGY=-651.13186278<br>Sn -0.0000000000 0.0000000000 0.6479141070<br>C 0.0000000000 0.0000000000 2.7714508625<br>H -1.6285455322 0.0000000000 0.2138356104<br>H 0.8142727661 1.4103618021 0.2138356103<br>H 0.8142727661 -1.4103618021 0.2138356103<br>P -0.0000000000 0.0000000000 -3.0138936226<br>H 1.1982175697 0.0000000000 -3.7599270811<br>H -0.5991087849 -1.0376868546 -3.7599270811<br>H -0.5991087849 1.0376868546 -3.7599270811

	N -0.0000000000 0.0000000000 3.9336506884
$NCSnH_3\cdots NCH$	CCSD(T)-F12C/USERDEF ENERGY=-401.73061482<br>Sn 0.0000208300 0.0000000000 0.3571657682<br>C -0.0000191206 0.0000000000 2.4889290650<br>H 1.6340870640 0.0000000000 -0.0523250764<br>H -0.8169904039 -1.4151162456 -0.0523541946<br>H -0.8169904039 1.4151162456 -0.0523541946<br>N -0.0000018235 0.0000000000 -2.6835333181<br>C -0.0000195210 0.0000000000 -3.8370167113<br>H -0.0000344703 0.0000000000 -4.9046068516<br>N -0.0000574424 0.0000000000 3.6513639880
$NCSnH_3\cdot\cdot H_2CCH_2$	CCSD(T)-F12C/USERDEF ENERGY=-386.88666330<br>Sn 0.0135900326 1.0076092904 0.0000000000<br>C 0.0169582293 3.1281554705 0.0000000000<br>N 0.0205420567 4.2902764504 0.0000000000<br>H -1.6148618839 0.5703573492 0.0000000000<br>H 0.8260235610 0.5630447812 1.4086558188<br>C -0.0229768406 -2.4776915841 0.6673738542<br>C -0.0229768406 -2.4776915841 -0.6673738542<br>H -0.9415899061 -2.3755478252 1.2311731377<br>H 0.8933957344 -2.5880987089 1.2325500188<br>H 0.8933957344 -2.5880987089 -1.2325500188<br>H -0.9415899061 -2.3755478252 -1.2311731377<br>H 0.8260235610 0.5630447812 -1.4086558188
$NCSnH_3\cdots OH_2$	CCSD(T)-F12C/USERDEF ENERGY=-384.79114744<br>Sn 0.0877082854 0.0066347631 0.0000000000<br>C -2.0458016277 -0.0105049664 0.0000000000<br>N -3.2082296139 -0.0151163959 0.0000000000<br>H 0.4957176976 -0.8028731547 1.4199576322<br>H 0.4957176976 -0.8028731547 -1.4199576322<br>H 0.4656878369 1.6492500646 0.0000000000<br>O 2.9542012087 -0.0623661546 0.0000000000<br>H 3.4974249037 0.1502133653 -0.7613151179<br>H 3.4974249037 0.1502133653 0.7613151179
$NCSnH_3\cdots NH_3$	CCSD(T)-F12C/USERDEF ENERGY=-364.92759905<br>Sn -0.0000000000 0.0000000000 -0.2396799549<br>C -0.0000000001 0.0000000000 1.9136535848<br>H -1.6520207471 0.0000000000 -0.5777335010<br>H 0.8260103737 1.4306919347 -0.5777335011<br>H 0.8260103737 -1.4306919347 -0.5777335011<br>N -0.0000000000 0.0000000000 -3.0235428140<br>H 0.9383703269 0.0000000000 -3.4058855486<br>H -0.4691851634 -0.8126525412 -3.4058855486<br>H -0.4691851634 0.8126525412 -3.4058855486<br>N -0.0000000000 0.0000000000 3.0766768116
$NCSnH_3\cdots CNH$	CCSD(T)-F12C/USERDEF ENERGY=-401.70705839<br>Sn 0.0000000000 0.0000000000 0.4157659517<br>C -0.0000000000 0.0000000000 2.5503689164<br>H 1.6351791358 0.0000000000 0.0109140614<br>H -0.8175895679 -1.4161066713 0.0109140615<br>H -0.8175895679 1.4161066713 0.0109140615

	C 0.0000000000 0.0000000000 -2.7276725668<br>N -0.0000000000 0.0000000000 -3.8948592259<br>H 0.0000000000 0.0000000000 -4.8923549644<br>N 0.0000000000 0.0000000000 3.7128804126
$NCSnH_3$···NP	CCSD(T)-F12C/USERDEF ENERGY=-704.00540454<br>Sn -0.0000000001 0.0000000000 1.0527294428<br>C -0.0000000004 0.0000000000 3.1907296657<br>H 1.6386179112 0.0000000000 0.6623331057<br>H -0.8193089555 -1.4190847385 0.6623331055<br>H -0.8193089555 1.4190847385 0.6623331055<br>N 0.0000000000 0.0000000000 -1.8940288057<br>P -0.0000000001 0.0000000000 -3.3832816888<br>N 0.0000000003 0.0000000000 4.3533611389
$NCSnH_3$···CS	CCSD(T)-F12C/USERDEF ENERGY=-744.14547830<br>Sn -0.0000033882 0.0000000000 1.1569528901<br>C -0.0000027071 0.0000000000 3.2891030111<br>H 1.6340842523 0.0000000000 0.7462141831<br>H -0.8170301930 -1.4151425803 0.7462275717<br>H -0.8170301930 1.4151425803 0.7462275717<br>C -0.0000049897 0.0000000000 -1.9888395679<br>S -0.0000026004 0.0000000000 -3.5199226354<br>N -0.0000101810 0.0000000000 4.4515585933

Table S4. Optimised geometries (Å) and energies (au) of ZSi···B complexes calculated at the CCSD(T)(F12c)/cc-pVDZ-F12 level. Z= O and S; B= N2, CO, HCCH, PH3, HCN, H2CCH2, HNC, H2O, NH3, CS, NP

OSi···B complexes.

OSi···N_2	CCSD(T)-F12C/CC-PVDZ-F12 ENERGY=-473.63555535<br>Si 0.0000000000 -0.3634503514 -1.8371393591<br>O 0.0000000000 -1.4030273653 -0.7363241588<br>N 0.0000000000 0.6926667481 1.8295377117<br>N 0.0000000000 -0.0115707912 2.6731290917
OSi···CO	CCSD(T)-F12C/CC-PVDZ-F12 ENERGY=-477.41854659<br>Si 0.0000000000 -0.3232645185 -1.7754362685<br>O 0.0000000000 -1.4287081907 -0.7401238804<br>C 0.0000000000 0.6649897567 1.7649724034<br>O 0.0000000000 0.0016011927 2.6797910310
OSi···HCCH	CCSD(T)-F12C/CC-PVDZ-F12 ENERGY=-441.44158646<br>Si -0.4182950123 0.9767682935 0.5630833023<br>O -0.2435032338 2.3215327944 -0.1137822095<br>C -0.0104559489 -0.6535884403 -3.0685418770<br>C 0.0253918665 0.5260790916 -2.8183169162<br>H -0.0399417747 -1.6903597124 -3.3029504759<br>H 0.0522041033 1.5609679731 -2.5654918237
OSi···PH_3	CCSD(T)-F12C/CC-PVDZ-F12 ENERGY=-706.93802386<br>Si -0.0580358453 0.9165225856 0.3415275781<br>O -1.2375905622 1.4847895817 -0.4241160089<br>P 0.4433568886 0.0224434988 -2.9054047134<br>H 0.9066190530 -0.4824098007 -4.1415966645<br>H -0.8794382445 -0.4384861295 -3.0679370294<br>H 0.1748249974 1.3190509877 -3.3905194366
OSi···NCH	Not Converged
OSi··H_2CCH_2	CCSD(T)-F12C/CC-PVDZ-F12 ENERGY=-442.69247550<br>Si -0.6711636588 0.9840095242 0.3330749468<br>O 0.7912450957 1.3716990954 0.2444541216<br>C -0.0110007340 -0.4980464878 -2.7969164092<br>C -0.2485365644 0.7676144023 -3.1491300437<br>H 0.7648401002 -0.7411171116 -2.0818614396<br>H -0.5744235974 -1.3188692670 -3.2218373787<br>H -1.0134306801 1.0177410460 -3.8731765120<br>H 0.3261084755 1.5810801195 -2.7249255236
OSi···OH_2	CCSD(T)-F12C/CC-PVDZ-F12 ENERGY=-440.59899747<br>Si 0.3548572420 1.1479110746 -0.4530276975<br>O -1.1114376376 1.1661880356 -0.8503336384<br>O 0.4344138884 0.0569522539 -2.9767030083<br>H 0.4905210509 -0.7549387221 -3.4835719370<br>H -0.5010134714 0.1880978677 -2.7836513581
OSi···NH_3	CCSD(T)-F12C/CC-PVDZ-F12 ENERGY=-420.73472298<br>Si -0.0865378100 0.4419810587 -0.5025939347

	O -0.6259442005 1.8554892444 -0.7208473795<br>N 0.0312449338 0.0944568223 -2.8245324709<br>H 0.9417767772 -0.0064112529 -3.2580784198<br>H -0.5883533315 -0.5867462447 -3.2479388977<br>H -0.3224500820 1.0231410959 -3.0340551719
OSi···CNH	CCSD(T)-F12C/CC-PVDZ-F12 ENERGY=-457.51088796<br>Si 0.0000000000 -0.1827812518 -1.3930412870<br>O 0.0000000000 -1.6332434643 -0.9500610906<br>C 0.0000000000 0.6346324741 1.6180432670<br>N 0.0000000000 0.0961699611 2.6545110583<br>H 0.0000000000 -0.4107177191 3.5131580523
OSi···NP	CCSD(T)-F12C/CC-PVDZ-F12 ENERGY=-759.80978039<br>Si -0.4231933071 0.0000000000 -0.1783545640<br>O -0.0030960995 0.0000000000 -1.6363170814<br>N 2.4101235280 0.0000000000 0.6422943425<br>P 3.5300758787 0.0000000000 -0.3415326971
OSi···CS	CCSD(T)-F12C/CC-PVDZ-F12 ENERGY=-799.95001980<br>Si -0.5575958058 0.0000000000 -0.1781133444<br>O -0.0742421223 0.0000000000 -1.6161034590<br>C 2.4107039704 0.0000000000 0.5255562129<br>S 3.7350439578 0.0000000000 -0.2452494095

SSi···B optimised complexes

SSi···N_2	CCSD(T)-F12C/CC-PVDZ-F12 ENERGY=-796.23005343<br>Si -0.7102203196 0.5133600058 0.0000000000<br>S -0.1053974176 2.3519652634 0.0000000000<br>N 2.9613731368 -0.3317192956 0.0000000000<br>N 3.9714014071 0.1013123337 0.0000000000
SSi···CO	CCSD(T)-F12C/CC-PVDZ-F12 ENERGY=-800.01280944<br>Si -0.7093723739 0.5118469916 0.0000000000<br>S -0.0899628652 2.3463138457 0.0000000000<br>C 2.9434971287 -0.3445686229 0.0000000000<br>O 3.9729949171 0.1213260929 0.0000000000
SSiH···HCCH	CCSD(T)-F12C/CC-PVDZ-F12 ENERGY=-764.03475001<br>Si -0.4215038190 0.6348912155 0.4407101076<br>S -0.2931146270 2.5670798512 0.3484888759<br>C -0.0150724851 -0.6421749044 -3.0315260808<br>C 0.0554039290 0.5616753402 -3.0184450770<br>H -0.0763422628 -1.7038182757 -3.0495296183<br>H 0.1160292649 1.6237467732 -2.9956982072
SSi···PH_3	CCSD(T)-F12C/CC-PVDZ-F12 ENERGY=-1029.53204008<br>Si -0.5235916176 0.4790459760 -0.1303615415<br>S 0.2465777286 2.1810394414 -0.6545913840<br>P -0.2744941170 -0.2316823592 -3.5628233497<br>H 1.0575170341 0.2203488229 -3.6808043755<br>H -0.3143397897 -0.8204132096 -4.8477298219<br>H -0.8421483693 0.9929061018 -3.9758871839
SSi···NCH	CCSD(T)-F12C/CC-PVDZ-F12 ENERGY=-780.12814323<br>Si -0.2863350327 0.4753234627 0.0000000000<br>S 0.1497348343 2.3653342372 0.0000000000

	N 2.7534774145 -0.5902516011 0.0000000000<br>C 3.7783765091 -0.0576368618 0.0000000000<br>H 4.7219030816 0.4421490703 0.0000000000
SSi··H_2CCH_2	Si -0.4633772767 0.6394538010 0.4992927832<br>S -0.0002553220 2.5209035264 0.5110463917<br>C -0.0113494302 -0.6663812965 -3.0270342765<br>C -0.0461436568 0.6669261597 -3.0660148568<br>H 0.9066195144 -1.2010736779 -2.8193814535<br>H -0.8986774043 -1.2602372325 -3.2041650455<br>H -0.9628568806 1.2023816354 -3.2767982412<br>H 0.8396788930 1.2621384052 -2.8872635397
SSi···OH_2	CCSD(T)-F12C/CC-PVDZ-F12 ENERGY=-763.19043732<br>Si -0.8780042516 0.4897245066 -0.2965058005<br>S 0.3900666871 1.9553003835 -0.4812026107<br>O -0.0944992111 -0.5317368435 -2.6885997242<br>H 0.4437672784 0.2464688344 -2.8603352490<br>H -0.5113744745 -0.7509573666 -3.5248500720
SSi···NH_3	CCSD(T)-F12C/CC-PVDZ-F12 ENERGY=-743.32949649<br>Si -0.4167152015 0.2113116920 -0.6652890586<br>S -0.0480520873 2.1489721507 -0.5527365913<br>N -0.0986149123 0.0825510555 -2.8307473504<br>H 0.8783493474 0.3041742115 -2.9950288372<br>H -0.3271786986 -0.7744222074 -3.3237569198<br>H -0.6380521607 0.8493238212 -3.2204875173
SSi···CNH	CCSD(T)-F12C/CC-PVDZ-F12 ENERGY=-780.10436180<br>Si -0.3724883755 0.4715952144 0.0000000000<br>S -0.0038348791 2.3766962251 0.0000000000<br>C 2.7749667411 -0.4173670404 0.0000000000<br>N 3.8887329107 -0.0625696828 0.0000000000<br>H 4.8297804096 0.2665635910 0.0000000000
SSi···NP	CCSD(T)-F12C/CC-PVDZ-F12 ENERGY=-1082.40256975<br>Si -0.3439789962 0.4233308823 0.0000000000<br>S -0.0591628425 2.3448800144 0.0000000000<br>N 2.5684897140 -0.3451425798 0.0000000000<br>P 3.9518089314 0.2118499903 0.0000000000
SSi···CS	CCSD(T)-F12C/CC-PVDZ-F12 ENERGY=-1122.56859358<br>Si -0.0447250692 0.0000000000 -0.3062077118<br>S 0.5884053793 0.0000000000 1.7364743619<br>C 1.6961429046 0.0000000000 0.3339551571<br>S 3.2979767853 0.0000000000 0.1705781929

Table S5. Optimised geometries (Å) and energies (au) of ZGe···B complexes calculated at the CCSD(T)(F12c)/cc-pVDZ-F12 level. Z= O and S; B= N2, CO, HCCH, PH3, HCN, H2CCH2, HNC, H2O, NH3, CS, NP

OGe···N_2	CCSD(T)-F12C/USERDEF ENERGY=-478.06878760<br>Ge 0.0000000000 -0.2092795741 -1.7362078046<br>O 0.0000000000 -1.6276181002 -0.9049307264<br>N 0.0000000000 0.6181415180 1.7920717224<br>N 0.0000000000 0.1333741563 2.7782708085
OGe···CO	CCSD(T)-F12C/USERDEF ENERGY=-481.85184108<br>Ge 0.0000000000 -0.1965549332 -1.7056791015<br>O 0.0000000000 -1.6338433489 -0.9068212255<br>C 0.0000000000 0.6066878557 1.7569137621<br>O 0.0000000000 0.1383284263 2.7847905648
OGeH···HCCH	CCSD(T)-F12C/USERDEF ENERGY=-445.87525632<br>Ge 0.7192834111 0.7032940812 0.0000000000<br>O -0.9121659240 0.9216054109 0.0000000000<br>C -0.8404971860 -2.3457491059 0.0000000000<br>C 0.0257699706 -3.1862545156 0.0000000000<br>H 0.7748399409 -3.9411981467 0.0000000000<br>H -1.5794156127 -1.5771222939 0.0000000000
OGe···PH_3	CCSD(T)-F12C/USERDEF ENERGY=-711.37174292<br>Ge 0.0000000000 -0.5854281222 -1.6017081344<br>O 0.0000000000 -1.5014535514 -0.2322263665<br>P 0.0000000000 1.5458942749 1.0670602688<br>H 0.0000000000 2.6599315073 1.9371468280<br>H -1.0382500759 0.8613461214 1.7308543849<br>H 1.0382500759 0.8613461214 1.7308543849
OGe···NCH	CCSD(T)-F12C/USERDEF ENERGY=-461.97469457<br>Ge 0.0000000000 -1.7472945651 -1.7235490714<br>O 0.0000000000 -0.9983237466 -0.2584332421<br>N 0.0000000000 0.9369284853 3.4781317159<br>C 0.0000000000 0.4046052911 2.4517989861<br>H 0.0000000000 -0.0918554646 1.4946616112
OGe··H_2CCH_2	CCSD(T)-F12C/USERDEF ENERGY=-447.12596873<br>Ge 0.3487136591 0.9878745684 0.0000000000<br>O -1.2967760389 1.0059499011 0.0000000000<br>H -0.8144439424 -1.9175668325 1.2243102725<br>C -0.0473673688 -2.4407754973 0.6677093436<br>C -0.0473673688 -2.4407754973 -0.6677093436<br>H -0.8144439424 -1.9175668325 -1.2243102725<br>H 0.7082497411 -2.9718439799 -1.2322577835<br>H 0.7082497411 -2.9718439799 1.2322577835
OGe···OH_2	CCSD(T)-F12C/USERDEF ENERGY=-445.03347762<br>Ge -0.0553709306 0.1238033071 -1.1311500183<br>O -0.0120966082 -1.3176625952 -0.3260179184<br>O 0.0837559828 0.8382127424 1.4978285687<br>H 0.1459323658 -0.1259099799 1.4744799126<br>H 0.6762054302 1.1314863156 2.1922627454

OGe···NH_3	CCSD(T)-F12C/USERDEF ENERGY=-425.16830992<br>Ge 0.0000000000 0.0546830611 -0.9298993013<br>O 0.0000000000 -1.5040035676 -0.3626883737<br>N 0.0000000000 0.7647297115 1.4699544111<br>H 0.0000000000 -0.2192519900 1.7226114373<br>H 0.8167887545 1.1980986170 1.8839923762<br>H -0.8167887545 1.1980986170 1.8839923762
OGe···CNH	CCSD(T)-F12C/USERDEF ENERGY=-461.94458644<br>Ge 0.0000000000 -0.1258808792 -1.3860612841<br>O 0.0000000000 -1.6855982524 -0.8561841733<br>C 0.0000000000 0.6855273625 1.6148710254<br>N 0.0000000000 0.0925273189 2.6208379441<br>H 0.0000000000 -0.4625055497 3.4491464879
OGe···NP	CCSD(T)-F12C/USERDEF ENERGY=-764.24371855<br>Ge 0.0000000000 -0.1083476447 -1.3319313178<br>O 0.0000000000 -1.6629857512 -0.7823173005<br>N 0.0000000000 0.8208109340 1.4498092855<br>P 0.0000000000 -0.1348595382 2.5936433326
OGe···CS	CCSD(T)-F12C/USERDEF ENERGY=-804.38375348<br>Ge 0.0000000000 -0.1174473904 -1.4717406492<br>O 0.0000000000 -1.6348166619 -0.8279929697<br>C 0.0000000000 0.6949893164 1.4394710924<br>S 0.0000000000 -0.0281072639 2.7894665263

SGe···N_2	CCSD(T)-F12C/USERDEF ENERGY=-800.69239484<br>Ge 0.0000000000 -0.0658496410 -1.7011368880<br>S 0.0000000000 -2.0494095558 -1.2716383794<br>N 0.0000000000 0.6961320368 1.9322317013<br>N 0.0000000000 0.3337451600 2.9697475662
SGe···CO	CCSD(T)-F12C/USERDEF ENERGY=-804.47525046<br>Ge 0.0000000000 -0.0531615476 -1.7188253982<br>S 0.0000000000 -2.0129279074 -1.1878443117<br>C 0.0000000000 0.6964667758 1.8919773171<br>O 0.0000000000 0.2842406789 2.9438963928
SGeH···HCCH	CCSD(T)-F12C/USERDEF ENERGY=-768.49611424<br>Ge -0.3517085924 0.0000000000 -0.9194775545<br>S 1.6114809942 0.0000000000 -1.4402991829<br>C -0.0796248097 0.6029078741 2.6889271154<br>C -0.0796248097 -0.6029078741 2.6889271154<br>H -0.0720111142 -1.6664811909 2.6951901544<br>H -0.0720111142 1.6664811909 2.6951901544
SGe···PH_3	CCSD(T)-F12C/USERDEF ENERGY=-1033.99492616<br>Ge 0.0000000000 -0.4526432395 -1.6892311315<br>S 0.0000000000 -1.9235147129 -0.2812289861<br>P 0.0000000000 1.6049040064 1.0971596646<br>H 0.0000000000 2.7382440493 1.9420880568<br>H -1.0373239236 0.9373231236 1.7815968817<br>H 1.0373239236 0.9373231236 1.7815968817
SGe···NCH	CCSD(T)-F12C/USERDEF ENERGY=-784.59114376<br>Ge 0.0000000000 0.0119652492 -1.3658527714

	S 0.0000000000 -1.9982743859 -1.0496440886<br>N 0.0000000000 0.8340184903 1.7155119849<br>C 0.0000000000 0.1483402553 2.6443885581<br>H 0.0000000000 -0.4919896088 3.4982063169
SGe··H_2CCH_2	CCSD(T)-F12C/USERDEF ENERGY=-769.74927355<br>Ge 0.4704106318 1.0041115247 0.0000000000<br>S -1.5410846395 1.2965658856 0.0000000000<br>H -0.7928549576 -1.9683660526 1.2277160873<br>C -0.0293481221 -2.4930428256 0.6675366614<br>C -0.0293481221 -2.4930428256 -0.6675366614<br>H -0.7928549576 -1.9683660526 -1.2277160873<br>H 0.7278113478 -3.0226315566 -1.2313867010<br>H 0.7278113478 -3.0226315566 1.2313867010
SGe···OH_2	CCSD(T)-F12C/USERDEF ENERGY=-767.65404118<br>Ge -0.0073214905 0.1327803830 -1.0641007033<br>S -0.0736171703 -1.8354370728 -0.5161606771<br>O 0.0589311884 0.8082519390 1.5442178330<br>H 0.1383685944 -0.1253598379 1.7649819627<br>H 0.7073314486 1.2634106305 2.0859510291
SGe···NH_3	CCSD(T)-F12C/USERDEF ENERGY=-747.79102611<br>Ge 0.0000000000 0.2255340641 -0.8695450637<br>S 0.0000000000 -1.8053983634 -0.5091955789<br>N 0.0000000000 0.7662387436 1.4923100903<br>H 0.0000000000 -0.1836068669 1.8516774980<br>H 0.8181582658 1.2447934361 1.8513579885<br>H -0.8181582658 1.2447934361 1.8513579885
SGe···CNH	CCSD(T)-F12C/USERDEF ENERGY=-784.56737362<br>Ge 0.0000000000 0.0304799909 -1.4464357723<br>S 0.0000000000 -1.9896210758 -1.1903696046<br>C 0.0000000000 0.6433204665 1.6954137456<br>N 0.0000000000 0.1394865769 2.7495240605<br>H 0.0000000000 -0.3196059584 3.6344775706
SGe···NP	CCSD(T)-F12C/USERDEF ENERGY=-1086.86580513<br>Ge 0.0000000000 0.0238827386 -1.3621534036<br>S 0.0000000000 -2.0023044626 -1.1387993898<br>N 0.0000000000 0.6871605299 1.5097119121<br>P 0.0000000000 0.2058791941 2.9204448814
SGe···CS	CCSD(T)-F12C/USERDEF ENERGY=-1127.00663613<br>Ge 0.0000000000 0.0230425203 -1.4800492002<br>S 0.0000000000 -1.9697558131 -1.0579873507<br>C 0.0000000000 0.7280982639 1.5270975369<br>S 0.0000000000 0.1332330287 2.9401430141

Table S6. Optimised geometries (Å) and energies (au) of ZSn···B complexes calculated at the CCSD(T)(F12c)/cc-pVDZ-F12 level. Z= O and S; B= N2, CO, HCCH, PH3, HCN, H2CCH2, HNC, H2O, NH3, CS, NP

OSn···N_2	CCSD(T)-F12C/USERDEF ENERGY=-398.48657745<br>Sn 0.0000000000 -0.1520395854 -1.7835728196<br>O 0.0000000000 -1.7005250397 -0.8136758268<br>N 0.0000000000 0.6456612403 1.8027896620<br>N 0.0000000000 0.2052599861 2.8094872110
OSn···CO	CCSD(T)-F12C/USERDEF ENERGY=-402.26995516<br>Sn 0.0000000000 -0.2074627285 -1.8597261212<br>O 0.0000000000 -1.4993504780 -0.5674985864<br>C 0.0000000000 0.6849268494 1.7643909911<br>O 0.0000000000 0.0202433571 2.6778627166
OSnH···HCCH	CCSD(T)-F12C/USERDEF ENERGY=-366.29534501<br>Sn 0.2130018310 2.5722008199 0.9116324802<br>O 0.3853987272 0.8000640072 0.5025418746<br>C 0.6816755141 -2.2509072464 -0.2013913581<br>C 0.7942577923 -3.4215626132 -0.4768156105<br>H 0.8933771937 -4.4518989909 -0.7192426929<br>H 0.5816649419 -1.2111379768 0.0432753068
OSn···PH_3	CCSD(T)-F12C/USERDEF ENERGY=-631.79115826<br>Sn 0.0000000000 -0.4276911682 -1.6070302884<br>O 0.0000000000 -1.4922446906 -0.1141571570<br>P 0.0000000000 1.4694241216 1.0864886376<br>H 0.0000000000 2.6037039414 1.9307817839<br>H -1.0436863262 0.7904378979 1.7427980119<br>H 1.0436863262 0.7904378979 1.7427980119
OSn···NCH	CCSD(T)-F12C/USERDEF ENERGY=-382.39471239<br>Sn 0.0000000000 -1.7218958400 -1.8586409843<br>O 0.0000000000 -0.9257400553 -0.2131848710<br>N 0.0000000000 0.8743828855 3.5247647002<br>C 0.0000000000 0.3731375377 2.4825547745<br>H 0.0000000000 -0.0958245282 1.5071163807
OSn··H_2CCH_2	CCSD(T)-F12C/USERDEF ENERGY=-367.54457755<br>Sn 0.6378303015 -0.2091544226 0.0000000000<br>O 0.4039542437 1.6050579297 0.0000000000<br>H -3.2161951462 -0.8566789490 1.2338310081<br>C -2.7579281842 -0.0553395643 0.6680884685<br>C -2.7579281842 -0.0553395643 -0.6680884685<br>H -3.2161951462 -0.8566789490 -1.2338310081<br>H -2.3103663776 0.7604204070 -1.2219521389<br>H -2.3103663776 0.7604204070 1.2219521389
OSn···OH_2	CCSD(T)-F12C/USERDEF ENERGY=-365.45563653<br>Sn -0.0086903521 0.1029654346 -1.0545323882<br>O -0.0033482666 -1.4636309878 -0.0855861782<br>O 0.0306888606 0.7739190993 1.4356776720<br>H 0.0678907169 -0.2030368813 1.4422338254<br>H 0.7356788007 1.0858575027 2.0076945580

OSn···NH_3	CCSD(T)-F12C/USERDEF ENERGY=-345.59078877<br>Sn 0.0000000000 0.1421505574 -0.9881985956<br>O 0.0000000000 -1.5464620286 -0.2439804447<br>N 0.0000000000 0.7717754720 1.4614067367<br>H 0.0000000000 -0.2325621124 1.6395680593<br>H 0.8177920899 1.1787262806 1.8995835837<br>H -0.8177920899 1.1787262806 1.8995835837
OSn···CNH	CCSD(T)-F12C/USERDEF ENERGY=-382.36390312<br>Sn 0.0000000000 -0.0224503232 -1.3657032818<br>O 0.0000000000 -1.7313385295 -0.7045873439<br>C 0.0000000000 0.6649957515 1.5585266886<br>N 0.0000000000 0.0848768521 2.5703244636<br>H 0.0000000000 -0.4920337509 3.3840394734
OSn···NP	CCSD(T)-F12C/USERDEF ENERGY=-684.66385199<br>Sn 0.0000000000 -0.0018478421 -1.1848981914<br>O 0.0000000000 -1.7297530845 -0.5701259493<br>N 0.0000000000 0.7239345728 1.5129946304<br>P 0.0000000000 -0.0939766462 2.7570585103
OSn···CS	CCSD(T)-F12C/USERDEF ENERGY=-724.80367265<br>Sn 0.0000000000 0.0044850400 -1.2895907714<br>O 0.0000000000 -1.6692732053 -0.5349453356<br>C 0.0000000000 0.6143005478 1.4817602533<br>S 0.0000000000 -0.0511553825 2.8578048537

SSn···N_2	CCSD(T)-F12C/USERDEF ENERGY=-721.12797971<br>Sn 0.0000000000 0.0011782826 -1.7267434614<br>S 0.0000000000 -2.1389052833 -1.1822338607<br>N 0.0000000000 0.7277481279 1.9362814702<br>N 0.0000000000 0.4083358727 2.9877238519
SSn···CO	CCSD(T)-F12C/USERDEF ENERGY=-724.91101889<br>Sn 0.0000000000 0.0090504818 -1.7375197034<br>S 0.0000000000 -2.0861597049 -1.0373264177<br>C 0.0000000000 0.7299109415 1.8639572280<br>O 0.0000000000 0.3455552816 2.9259178930
SSnH···HCCH	CCSD(T)-F12C/USERDEF ENERGY=-688.93423445<br>Sn 0.4063182085 -0.5701223868 0.0000000000<br>S -1.7636309755 -1.0081200830 0.0000000000<br>C 0.4373673006 3.0950393625 0.0000000000<br>C -0.6894593833 2.6626633908 0.0000000000<br>H 1.4261168358 3.4872587571 0.0000000000<br>H -1.6767060060 2.2632912298 0.0000000000
SSn···PH_3	CCSD(T)-F12C/USERDEF ENERGY=-954.43209457<br>Sn 0.0000000000 -0.2803236403 -1.6672537733<br>S 0.0000000000 -1.9360869135 -0.1886558995<br>P 0.0000000000 1.5155679257 1.1169831075<br>H 0.0000000000 2.6923921453 1.9002240770<br>H -1.0432737938 0.8712592418 1.8101907426<br>H 1.0432737938 0.8712592418 1.8101907426
SSn···NCH	CCSD(T)-F12C/USERDEF ENERGY=-705.02824634<br>Sn 0.0000000000 0.1257513569 -1.3537128154

	S 0.0000000000 -2.0509865817 -0.9392655628<br>N 0.0000000000 0.8095809076 1.6719264357<br>C 0.0000000000 0.1296080220 2.6044972991<br>H 0.0000000000 -0.5098937047 3.4591646432
SSn··H_2CCH_2	CCSD(T)-F12C/USERDEF ENERGY=-690.18569956<br>Sn 0.6244590007 -0.3289156604 0.0000000000<br>S 0.7154641766 1.8826405832 0.0000000000<br>H -3.2315035576 -0.9008577109 1.2324483362<br>C -2.8087321824 -0.0794839168 0.6679825015<br>C -2.8087321824 -0.0794839168 -0.6679825015<br>H -3.2315035576 -0.9008577109 -1.2324483362<br>H -2.3933232130 0.7498327823 -1.2264310030<br>H -2.3933232130 0.7498327823 1.2264310030
SSn···OH_2	CCSD(T)-F12C/USERDEF ENERGY=-688.09370964<br>Sn 0.0262713975 0.2464474902 -1.0548448408<br>S -0.0515907469 -1.8572693162 -0.3128360960<br>O 0.0422190169 0.8205320376 1.4679265371<br>H 0.0765294147 -0.1332935588 1.6361160569<br>H 0.7287906770 1.2196575149 2.0091258319
SSn···NH_3	CCSD(T)-F12C/USERDEF ENERGY=-668.23133739<br>Sn 0.0000000000 0.2902742900 -0.9682267547<br>S 0.0000000000 -1.8839291708 -0.4219815642<br>N 0.0000000000 0.7955913345 1.4953168548<br>H 0.0000000000 -0.1849918495 1.7667617658<br>H 0.8186898143 1.2377049220 1.8980463132<br>H -0.8186898143 1.2377049220 1.8980463132
SSn···CNH	CCSD(T)-F12C/USERDEF ENERGY=-705.00478845<br>Sn 0.0000000000 0.1616491304 -1.3768320348<br>S 0.0000000000 -2.0324475148 -1.0427646415<br>C 0.0000000000 0.5956757074 1.6156687147<br>N 0.0000000000 0.1188514203 2.6803165914<br>H 0.0000000000 -0.3396687433 3.5662213703
SSn···NP	CCSD(T)-F12C/USERDEF ENERGY=-1007.30408287<br>Sn 0.0000000000 0.1372081091 -1.2059336167<br>S 0.0000000000 -2.0581412839 -0.8566834881<br>N 0.0000000000 0.6649472476 1.5394284910<br>P 0.0000000000 0.1543429271 2.9382176138
SSn···CS	CCSD(T)-F12C/USERDEF ENERGY=-1047.44468589<br>Sn 0.0000000000 0.1787579756 -1.2442034697<br>S 0.0000000000 -1.9884037331 -0.7161169968<br>C 0.0000000000 0.5913169254 1.4612155032<br>S 0.0000000000 0.1166858322 2.9141339632

Figure S1. Molecular electrostatic surface potentials of H_3MX (M = Si, Ge and Sn; X = H, F, Cl and CN) calculated at the MP2/aug-cc-pVTZ level of theory on the 0.001e/bohr^3 electron density iso-surface.

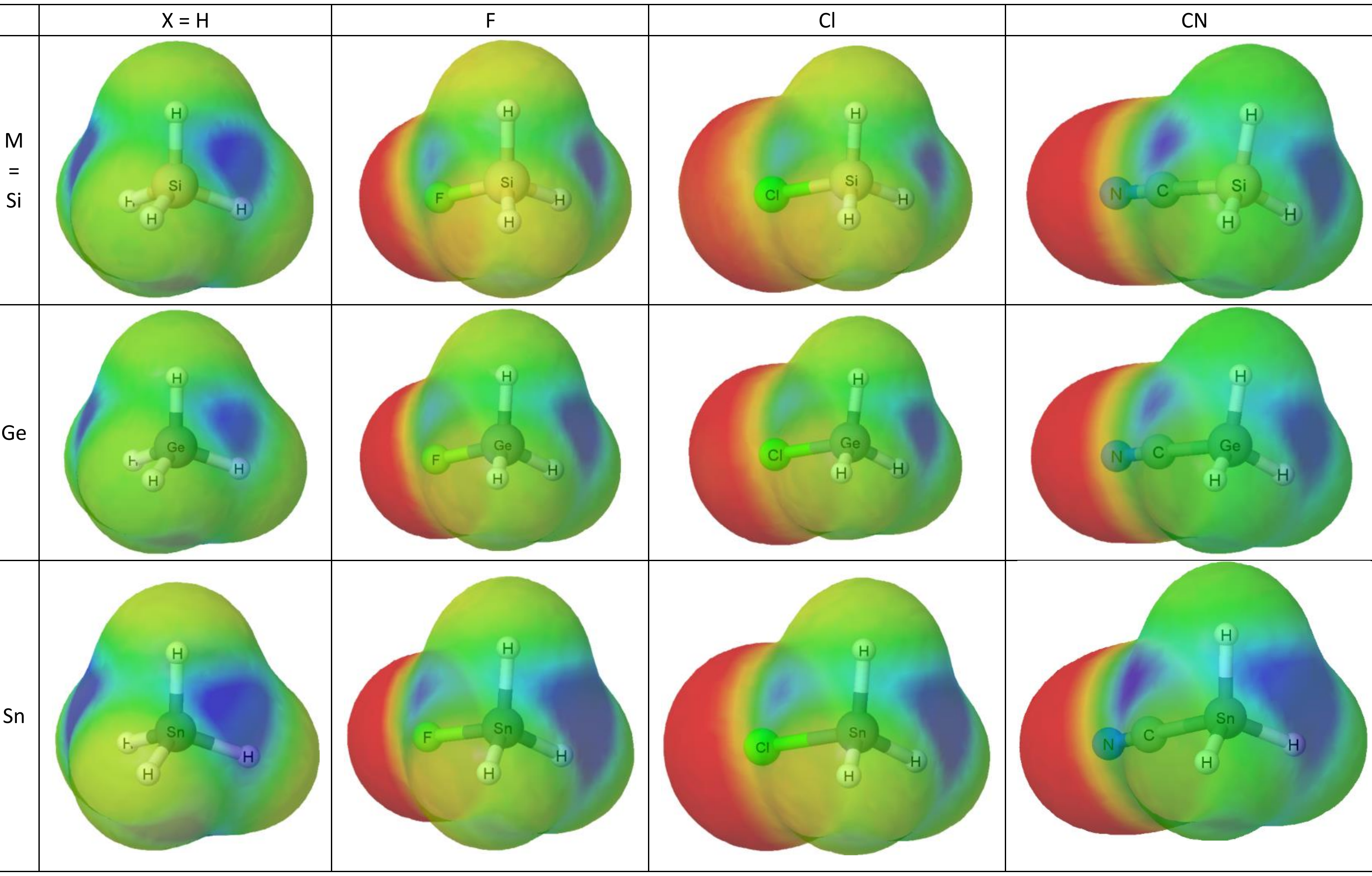

Color code:

MH_4: Red color < -40 kJ mol^{-1}, Blue color > 60 kJ mol^{-1}

XMH_3: Red color < -40 kJ mol^{-1} ,Blue color > 140 kJ mol^{-1}

Figure S2. Molecular electrostatic surface potentials of M-O and M-S (for M = Si, Ge and Sn) calculated at the MP2/aug-cc-pVTZ level of theory on the 0.001e/bohr^3 electron density iso-surface.

	X = O	S
M = Si	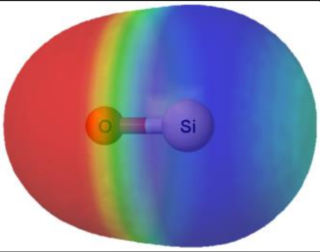	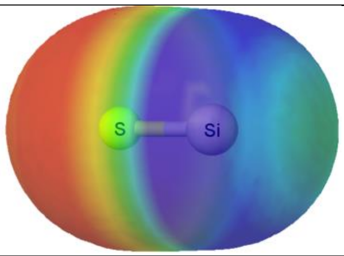
Ge	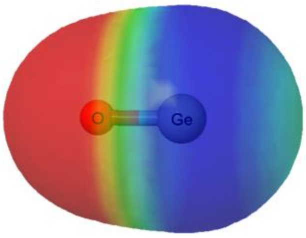	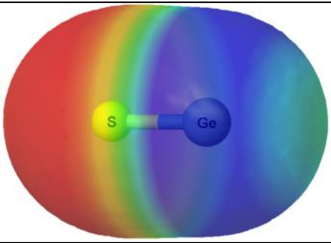
Sn	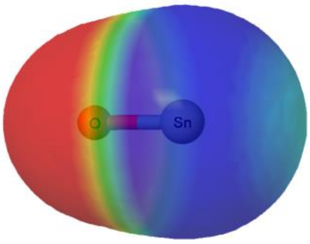	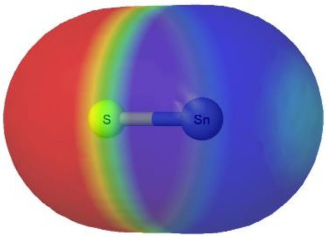

M-O: Red color < -40 kJ mol^{-1}, Blue color > 100 kJ mol^{-1}

M-S: Red color < -40 kJ mol^{-1} ,Blue color > 70 kJ mol^{-1}

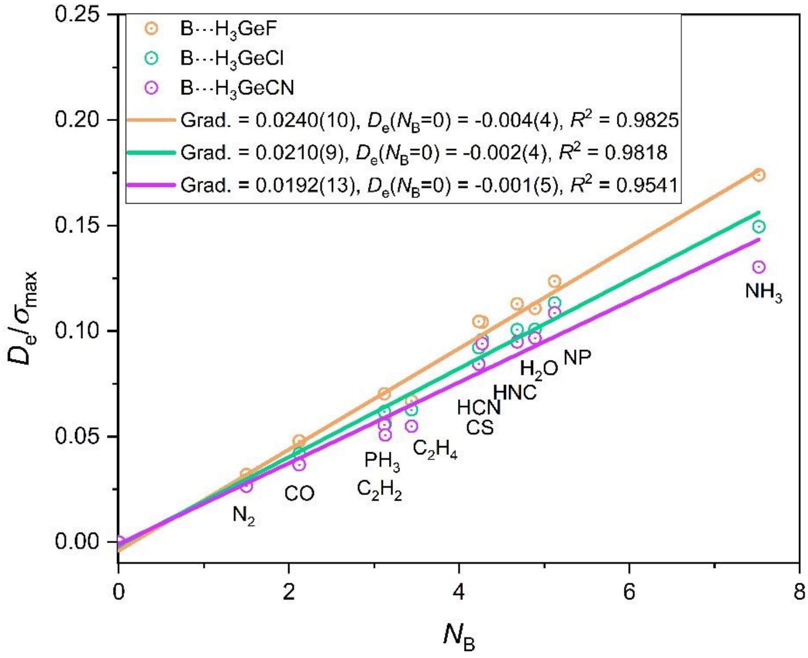

Figure S3 Graphs of $D_{\mathrm{e}}/\sigma_{\mathrm{max}}$ for the series of complexes B···H$_3$SGeX (X = F, Cl, CN) versus the nucleophilicities N_B of the Lewis bases B = N_2, CO, PH_3, C_2H_2, C_2H_4, HCN, HNC, CS, H_2O, NP and NH_3.

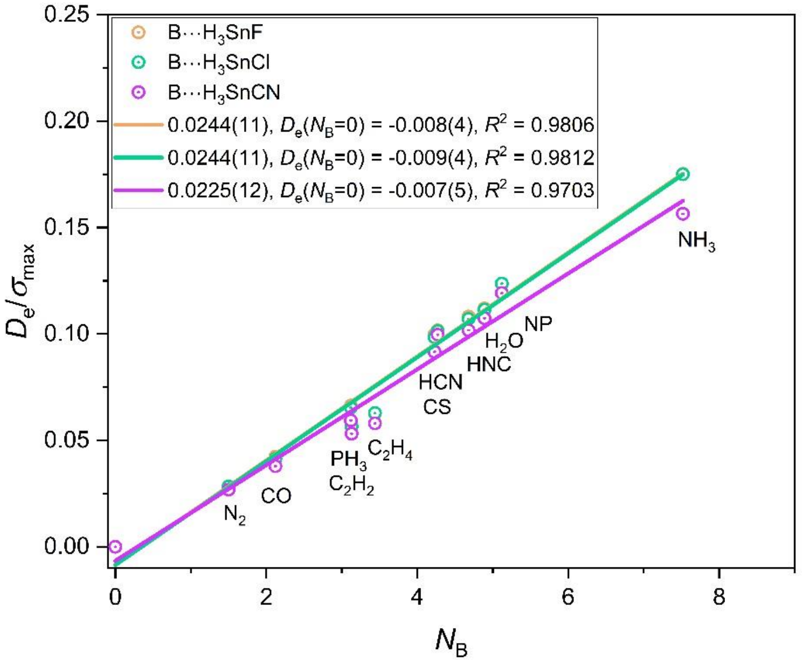

Figure S4 Graphs of $D_{\mathrm{e}}/\sigma_{\mathrm{max}}$ for the series of complexes BLH$_3$SnX (X = F, Cl, CN) versus the nucleophilicities N_B of the Lewis bases B = N_2, CO, PH_3, C_2H_2, C_2H_4, HCN, HNC, CS, H_2O, NP and NH_3.

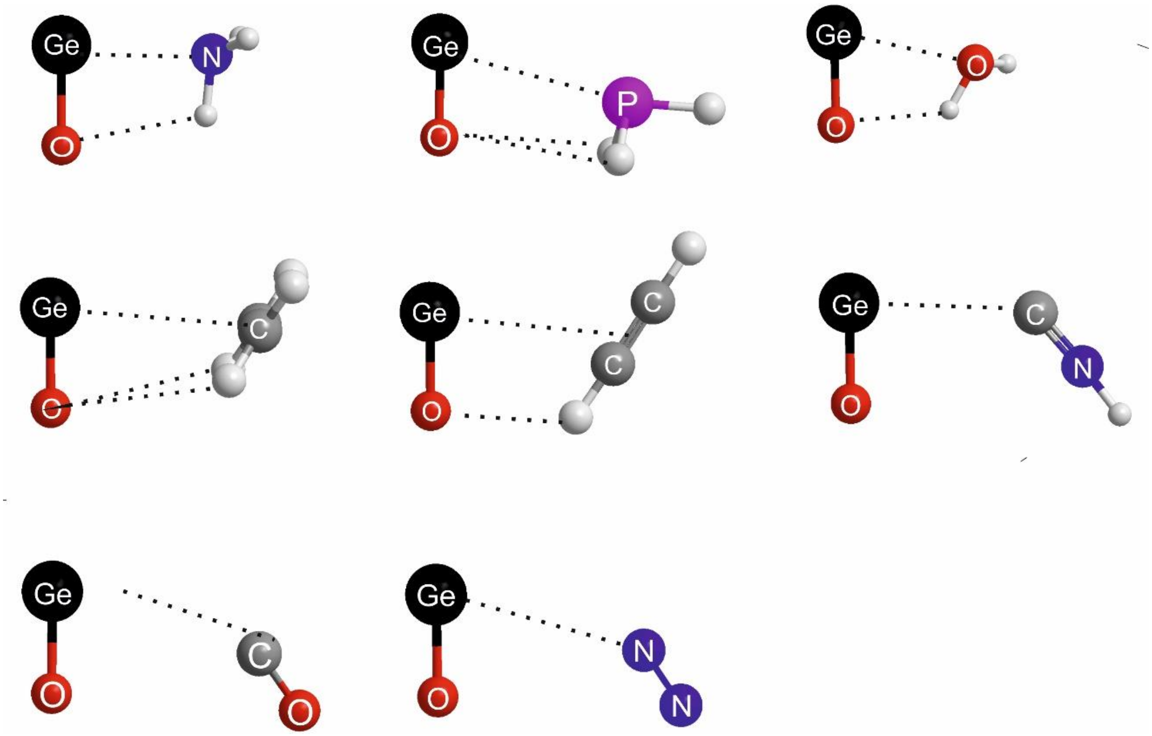

Figure S5. Representations of the geometries of several complexes BLGeO, as optimised at the CCSD(T)(F12c)/cc-pVDZ-F12 level of theory, with possible, significant non-covalent interaction indicated by dotted lines.

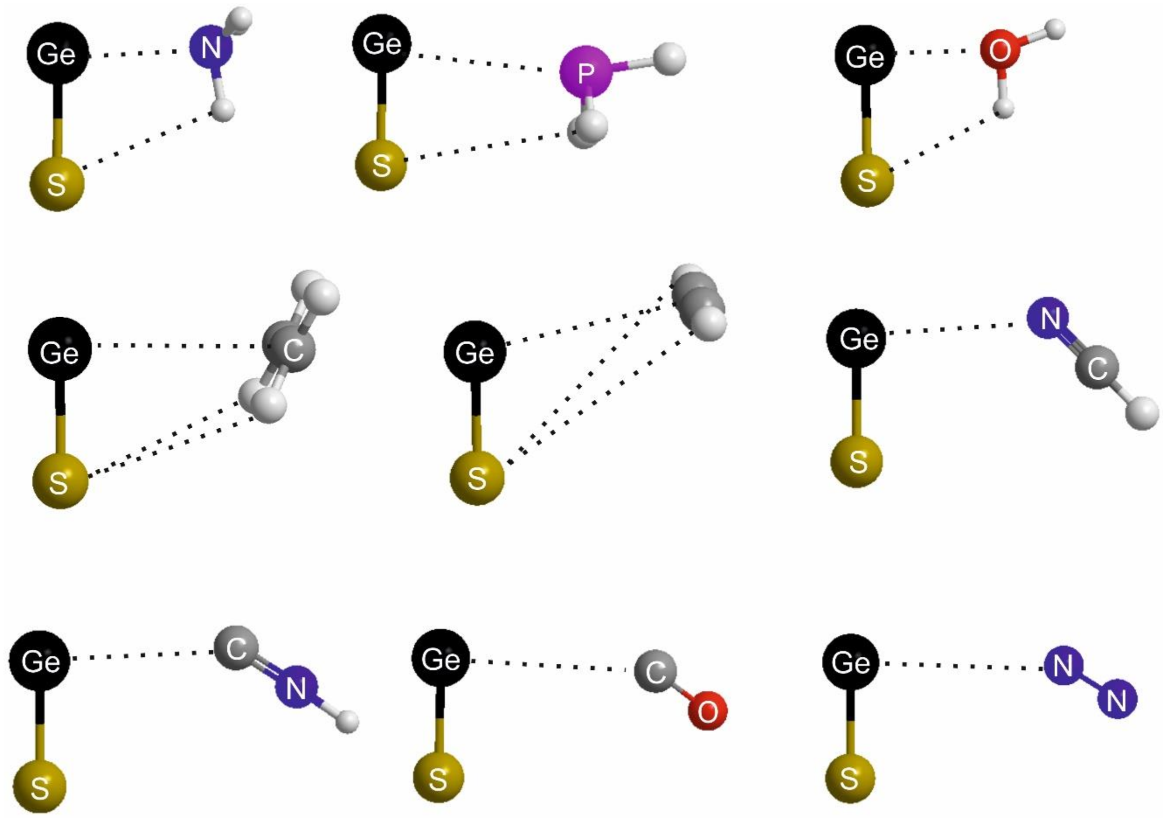

Figure S6. Representations of the geometries of several complexes B···GeS, as optimised at the CCSD(T)(F12c)/cc-pVDZ-F12 level of theory, with possible, significant non-covalent interaction indicated by dotted lines.
